# The AsiDNA™ decoy mimicking DSBs protects the normal tissue from radiation toxicity through a DNA-PK/p53/p21-dependent G1/S arrest

**DOI:** 10.1093/narcan/zcae011

**Published:** 2024-03-12

**Authors:** Anouk Sesink, Margaux Becerra, Jia-Ling Ruan, Sophie Leboucher, Maxime Dubail, Sophie Heinrich, Wael Jdey, Kristoffer Petersson, Charles Fouillade, Nathalie Berthault, Marie Dutreix, Pierre-Marie Girard

**Affiliations:** Institut Curie, Université PSL, CNRS UMR3347, INSERM U1021, 91405 Orsay, France; Université Paris-Saclay, CNRS UMR 3347, INSERM U1021, 91405 Orsay, France; Institut Curie, Université PSL, CNRS UMR3347, INSERM U1021, 91405 Orsay, France; Université Paris-Saclay, CNRS UMR 3347, INSERM U1021, 91405 Orsay, France; Oxford Institute for Radiation Oncology, Department of Oncology, University of Oxford, Old Road Campus Research Building, Roosevelt Drive, Oxford, UK; Histology platform, Institut Curie, CNRS UMR3348, 91405 Orsay, France; Institut Curie, Université PSL, CNRS UMR3347, INSERM U1021, 91405 Orsay, France; Université Paris-Saclay, CNRS UMR 3347, INSERM U1021, 91405 Orsay, France; Institut Curie, Université PSL, CNRS UMR3347, INSERM U1021, 91405 Orsay, France; Université Paris-Saclay, CNRS UMR 3347, INSERM U1021, 91405 Orsay, France; Valerio Therapeutics, 49 Bd du Général Martial Valin, 75015 Paris, France; Oxford Institute for Radiation Oncology, Department of Oncology, University of Oxford, Old Road Campus Research Building, Roosevelt Drive, Oxford, UK; Radiation Physics, Department of Hematology, Oncology and Radiation Physics, Skåne University Hospital, Lund University, Lund, Sweden; Institut Curie, Université PSL, CNRS UMR3347, INSERM U1021, 91405 Orsay, France; Université Paris-Saclay, CNRS UMR 3347, INSERM U1021, 91405 Orsay, France; Institut Curie, Université PSL, CNRS UMR3347, INSERM U1021, 91405 Orsay, France; Université Paris-Saclay, CNRS UMR 3347, INSERM U1021, 91405 Orsay, France; Institut Curie, Université PSL, CNRS UMR3347, INSERM U1021, 91405 Orsay, France; Université Paris-Saclay, CNRS UMR 3347, INSERM U1021, 91405 Orsay, France; Institut Curie, Université PSL, CNRS UMR3347, INSERM U1021, 91405 Orsay, France; Université Paris-Saclay, CNRS UMR 3347, INSERM U1021, 91405 Orsay, France

## Abstract

AsiDNA™, a cholesterol-coupled oligonucleotide mimicking double-stranded DNA breaks, was developed to sensitize tumour cells to radio- and chemotherapy. This drug acts as a decoy hijacking the DNA damage response. Previous studies have demonstrated that standalone AsiDNA™ administration is well tolerated with no additional adverse effects when combined with chemo- and/or radiotherapy. The lack of normal tissue complication encouraged further examination into the role of AsiDNA™ in normal cells. This research demonstrates the radioprotective properties of AsiDNA™. In vitro, AsiDNA™ induces a DNA-PK/p53/p21-dependent G1/S arrest in normal epithelial cells and fibroblasts that is absent in p53 deficient and proficient tumour cells. This cell cycle arrest improved survival after irradiation only in p53 proficient normal cells. Combined administration of AsiDNA™ with conventional radiotherapy in mouse models of late and early radiation toxicity resulted in decreased onset of lung fibrosis and increased intestinal crypt survival. Similar results were observed following FLASH radiotherapy in standalone or combined with AsiDNA™. Mechanisms comparable to those identified *in vitro* were detected both *in vivo*, in the intestine and ex vivo, in precision cut lung slices. Collectively, the results suggest that AsiDNA™ can partially protect healthy tissues from radiation toxicity by triggering a G1/S arrest in normal cells.

## Graphical Abstract

**Graphical Abstract:**
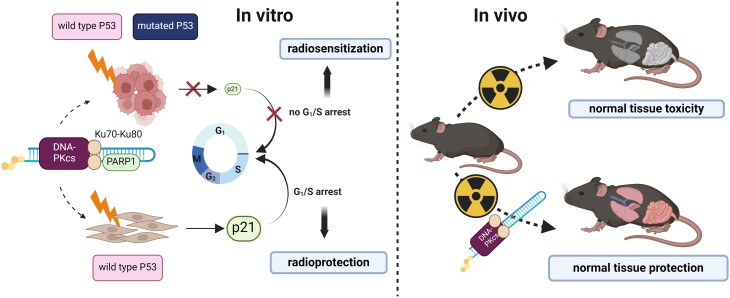

## Introduction

Radiotherapy and chemotherapy are customarily implemented in cancer treatments with curative intent; however, these therapies are often accompanied by the development of moderate to high levels of treatment-related toxicity. Radiotherapy frequently results in loss of epithelial integrity, tissue senescence, and cell death. Fibrosis formation, vascular damage with the potential development of secondary malignancies, and cardiac arrhythmia can all develop in the long term (1,2). Toxicities correlated to chemotherapy depend on the type of chemotherapeutic administered. These injuries range from anorexia, vomiting, and gastrointestinal toxicities to neurotoxicity (3). Consequently, treatment-induced toxicities often interfere with the completion of the initial treatment plan. To enhance treatment effect, it is crucial to alleviate treatment-related toxicities to both improve post-treatment outcomes and advance patients’ welfare. This can be achieved by expanding the therapeutic index using sensitizers or protective treatment modalities to shift the normal tissue complication probability or the tumour control probability (4). The DNA repair inhibitor AsiDNA™ has previously been validated as a suitable treatment agent to enhance this index. The active part of the molecule consists of two complementary oligonucleotides of 32 bases stabilized at one blunt end by a hexaethyleneglycol linker (5). The functionalization of a cholesterol group at the other blunt-end of the molecule allows its cellular uptake via LDL receptors expressed at the cell membrane both *in vitro* and *in vivo*. (6,7). AsiDNA was designed to mimic double-stranded breaks, triggering deceptive signalling of DNA damage and impairing DNA repair of chromosomes damaged by radiation or chemical treatments (8,9). Indeed, AsiDNA™ binds both DNA-dependent protein kinase (DNA-PK) and PARP enzymes, activating their kinase and polymerase activity, and consequently leading to modification of numerous proteins in the cell [see (10) and references therein]. The characteristic substrates phosphorylated by AsiDNA™-dependent DNA-PK activation are histone H2AX and heat shock protein 90 (HSP90) (6,9,11,12).

Several preclinical studies have demonstrated an additive or synergistic tumour control effect of AsiDNA™ combined with radiotherapy or chemotherapy, without any added toxicity. (10,13–17). These observations are further supported by *in vitro* data, revealing no additional toxicity after continuous or cycling treatment of AsiDNA™ on normal cell models, while simultaneously increasing tumour cell sensitivity with no acquired resistance (7,8,18). In addition, human clinical trials have failed to show any dose-limiting toxicity, with none reaching the maximum-tolerated dose (19,20). Recently, AsiDNA™ treatment in combination with carboplatin +/- paclitaxel was tested in patients bearing solid tumours (21). These case reports showed no increased toxicity of combined carboplatin and AsiDNA™ treatment. Moreover, combined treatment allowed the dose delivery times of carboplatin to be exceeded before the occurrence of toxicities (21). Taken together, these pre-clinical and clinical studies suggest that AsiDNA™ can increase the therapeutic window by radio- or chemo-sensitizing tumour cells upon treatment, while minimizing normal tissue injuries. However, the mechanism of normal tissue resistance remains still poorly understood.

To address this knowledge gap, in the present study, we aimed to characterize the molecular mechanism underlying the potential normal tissue protection capacities of AsiDNA™ and to demonstrate its radioprotective potential *in vivo*. To evaluate if the radioprotection property of AsiDNA™ is retained or enhanced with different modes of radiation, we combined AsiDNA™ treatment with conventional radiotherapy (CONV-RT) or FLASH radiotherapy (FLASH-RT). FLASH-RT is based on the delivery of dose rates over 1000 times higher (≥40 Gy/s) in comparison to CONV-RT (22). Numerous studies have demonstrated that FLASH-RT diminishes the severity of radiation-induced toxicities in normal tissues that remains present in CONV-RT, while maintaining an equivalent anti-tumour response (23–29).

Herein, we report that AsiDNA™ induces a DNA-PK/p53/p21-dependent G1/S arrest specifically in primary fibroblasts and immortalised epithelial cells, referred to as normal cells within this study, resulting in improved survival following ionizing radiation. This research provides evidence that this mechanism could account in mouse models for reduced early toxicity in the small intestine and reduced late toxicity in lung, demonstrating the potential benefit of the association of AsiDNA™ to standard radiotherapy in cancer treatment.

## Materials and methods

### Cell culture and transfection

Immortalized retinal pigment epithelial cell line hTERT (RPE-hTERT, kindly provided by A. Londono, Institut Curie, France), RPE-hTERT with shp53 (kindly provided by D. Fachinetti, Institut Curie, France), immortalized primary fibroblasts hTERT (VH10-hTERT, kindly provided by Aart G Jochemsen, and described in (30)), immortalized RPE-hTERT p21^−/−^ (kindly provided by R. G. Syljuåsen and described in (31)), primary human skin fibroblasts (BJ, ATCC CRL-2522), primary human lung fibroblasts (MRC-5, kindly provided by P. Jeggo, GDSC, Brighton, UK), and SV40-transformed MRC-5 fibroblasts (MRC-5v1, kindly provided by P. Jeggo, GDSC, Brighton, UK) were cultured in DMEM/F12 glutamax™ supplement medium (Thermo Fisher Scientific, France) supplemented with 10% fetal calf serum (FCS, Eurobio, France) and 100U/ml penicillin 100 μg/ml streptomycin (P/S, Thermo Fisher Scientific, France). A549 lung carcinoma cells (ATCC CCL-185), HCT116 colon carcinoma cells (ATCC CCL­247), U2OS osteosarcoma cells (ATCC HTB-96), and DAOY medulloblastoma cells (ATCC HTB­186) were cultured in DMEM/F12 glutamax™ supplement medium supplemented with 10% FCS, P/S and 1× Non-Essential Amino Acids (MEM NEAA 100X, Thermo Fisher Scientific, France). All cell lines were maintained in a humidified atmosphere at 37°C with 5% CO_2_. The absence of Mycoplasma contamination was determined in-house by using LookOut Mycoplasma PCR (Sigma-Aldrich). Transfection of cell lines are described in the Supplementary Materials and Methods.

### Molecules

AsiDNA™ (MW = 20931.4 g/mol) is a 64-nucleotide (nt) oligodeoxyribonucleotide consisting of two 32 nt strands of complementary sequence connected through a 1.19bis(phospho)-8-hydraza-2-hydroxy-4-oxa-9-oxo-nonadecane linker with cholesterol at the 5′-end and three phosphorothioate internucleotide linkages at each of the 5′ and the 3′ ends. The sequence is: 5′-X GsCsTs GTG CCC ACA ACC CAG CAA ACA AGC CTA GA L-CL TC TAG GCT TGT TTG CTG GGT TGT GGG CAC sAsGsC-3′, where L is an amino linker, X a cholesteryl tetraethylene glycol, CL a carboxylic (hydroxyundecanoic) acid linker, and s is a phosphorothioate linkage. AsiDNA™ was synthesized and purified by LGC (UK) and kindly provided by Wael Jdey (Valerio Therapeutics). The stock concentration of AsiDNA™ dissolved in water was at 40 mg/ml. Nol8 (MW = 6005.8 g/mol) has the same chemical structure as AsiDNA™ with the exception that it consists of two 8 nt strands of complementary sequence, and was synthesized and purified by Eurogentec (Belgium). The stock concentration of Nol8 dissolved in water was at 61 mg/ml.

### *In vitro* treatments

Cell culture medium was supplemented with AsiDNA™ at concentrations of 20 or 40 μmol/l 24 or 48 h prior to IR treatment. In vitro irradiation was conducted using the ElectronFLASH (S.I.T., Vicenza, Italy) at a dose rate of 0.4 Gy/s.

### Cell cycle analysis

Complete medium with total 10 μmol/l BrdU (Merck, France) was added to cells for a 40 min incubation either prior to- or post-AsiDNA™ treatment, under standard culture conditions. For drug treatment, cells were exposed to 1 μM Olaparib (AZD-2281, Roowin chemicals), 10 μM NU7026 (Merck, France), and 10 μM p21 inhibitor UC2288 (Merck, France) 1 h prior to AsiDNA™ treatment. Cells were harvested, fixed in cold 70% EtOH, and permeabilised in 1× PBS/0.5% BSA/0.1% Tween-20. BrdU detection was performed using FITC mouse anti-BrdU antibody (BD biosciences, France, #51-33284X). Following 1 h incubation, the cells were centrifuged and resuspended in 1× PBS containing 0.5% BSA, 10 μg/ml propidium iodide (Merck, France) and 0.2 mg/ml RNAse A (Merck, France). The data acquisition was performed using the LSRFortessa™ X-20 Cell Analyzer (BD biosciences, France), and the quantification of cell cycle performed using FlowJo (BD biosciences, France).

### Clonogenic survival after radiation

Cells were seeded at 1.5–3 × 10^5^ cells per 25 cm² flasks and 20 μmol/l AsiDNA were added for 24 h, prior to irradiation with doses of 0, 2, 4 and 6 Gy using the electronFLASH irradiator at a dose rate of 0.4 Gy/s. Following a recovery period of 24 h, the cells were trypsinized, counted and seeded for clonogenic survival. Eight to twelve days post seeding, cells were fixed and stained in 80% Methanol, 4% formaldehyde, 2.5% crystal violet. Colonies exceeding 50 cells were counted, and the obtained data was analysed using GraphPad Prism.

### Transfection

Prior to RNA interference, cells were attached overnight in 6-well plates for RPE-hTERT cells and in 60mm2 dishes for BJ cells. P53 siRNA; GAG UGG AAG GAA AUU UGC UGG A (20 nM, TP53HSS186390, Invitrogen), p21 siRNA; GAACUUCGACUUUGUCACCGAGACA (CDKN1, 40 nM, CDKNIAVHS40209, Invitrogen) or DNA-PK siRNA mix (PRKDC, 18 nM s773 GCGUUGGAGUGCUACAACATT, 18 nM s774 GCGCUUUUCUGGGUGAACUTT, ThermoFisher Scientific) were supplemented to the cells in Opti-MeM serum free medium (Gibco). RNA interference was performed following manufacturer's instructions for INTERFERin (Polyplus transfection) with medium replacement 7 h posttransfection. AsiDNA™ treatment on transfected cells was performed for 48 h starting at 24 h posttransfection.

### Western blot analysis

Cell pellets were lysed in lysis buffer [10 mM HEPES, pH 7.5, 100 mM NaCl, 300 mM sucrose, 3 mM MgCl_2_, 1 mM EGTA, 50 mM NaF, 20 mM ß-glycerophosphate, 0.3% Triton X-100, 0.1 mM sodium orthovanadate, and complete mini EDTA-free protease inhibitors (Roche Diagnosis)] on ice for 5 min. Following centrifugation at 240 rcf 4°C, supernatants were transferred into 1.5 ml Eppendorf tubes and protein concentration determined using Bradford assay (Bio-Rad). Twenty to thirty micrograms of protein extracts were separated on 4–15% Mini-PROTEAN® TGX™ Precast Protein Gels (Bio-Rad) and transferred onto PROTRAN® nitrocellulose membrane (Whatman) using a Mini Trans-Blot Cell (Bio-Rad). Membranes were probed overnight at 4°C with the following primary antibodies diluted in Intercept blocking buffer (LI-COR Biosciences – GmbH): anti-p53 (R&D systems, AF1355-sp, dil. 1:500), anti-p21 (Waf/Cip (12D1), Cell Signaling Technologies, 2947s, dil. 1:1000), anti-hsp90-p (T5/7, Cell Signaling Technologies, 3488s, dil. 1:1000), anti-DNA-PK (Thr2609, Novus Biologicals, dil. 1:1000), and anti-β-actin (Signa, A1978, dil. 1:2000). The membranes were probed with the appropriate secondary antibodies diluted in Intercept blocking buffer: IRDye 800CW goat anti rabbit (LI-COR 926-32211, dil. 1:15000), IRDye 680RD goat anti-mouse (LI-COR 926-32220, dil. 1:5000), IRDye 800CW goat anti mouse (LI-COR 926-32210, dil. 1:5000), IRDye 800CW donkey anti-goat (LICOR 926-32214, dil. 1:5000). Direct infrared fluorescence was detected on the Odyssey Infrared Imaging System (LI-COR Biosciences – GmbH).

### *Ex vivo* and *in vivo* experimentation

Studies were performed in accordance with the recommendations of the European Community (2010/63/UE) or UK Home Office guideline for the care and use of laboratory animals. Experimental procedures were explicitly approved by the ethics committee of Institut Curie CEEA-IC #118 (Authorization numberAPAFIS#5479-201605271 0291841 given by National Authority), or by the University of Oxford's Animal Welfare and Ethical Review Body (under project licenses PP8415318), in compliance with the international guidelines. All animals used within this research were acclimated for at least 1 week prior to experimentation. Mice were housed under pathogen-free conditions in cages containing sawdust with a maximum of six animals per cage, under a controlled 12 h light/dark cycle, a relative humidity of 55%, and a controlled temperature of 21°C. Food and sterile water were provided *ad libitum*. All experiments were conducted on C57BL/6J mice (Charles River, France) at 8–9 weeks of age, unless otherwise indicated in the corresponding materials and methods.

### Precision-cut lung slices (PCLS)

PCLS were obtained from the lungs of female C57BL/6J mice (Charles River, France) or male and female C57BL/6J p53 Knock-out mice (Curie collection), at 4–6 months old, as recently described (32), and briefly presented in the Supplementary Materials and Methods. AsiDNA™ or Nol8 treatment of 5 μmol/l was performed for 48 h in 24-well plates, followed by a 24 h co-incubation with 10 μmol/l EdU. EdU positive cells were revealed using EdU DetectPro Imaging kit Imaging (647 nm, BCK-EdUPro-IM647/BCK488-IV-IM-S, Baseclick), and visualized with the Inverted spinning disk-TIRF-FRAP (Nikon) with a 300 ms emission and 30% laser, DAPI (405 nm), 400 ms emission and 70% laser, 10× objective with 50 stacks of 3 μm. Data analysis was performed using IMARIS with spot function and PRISM software.

### Animal irradiation and fibrosis analysis

The female C57BL6/J mice model of radiation induced lung fibrosis was used (23,33). Mice were irradiated after 2 consecutive days of intraperitoneal AsiDNA™ injections (100 mg/kg), followed by a third day with intraperitoneal AsiDNA™ injection (200 mg/kg) and FLASH/CONV irradiation. Bilateral thorax irradiation of 13 Gy was performed using the ElectronFLASH (S.I.T., Vicenza, Italy), including a CONV dose rate of 0.4 Gy/s and a FLASH dose rate of >100 Gy/s (beam parameters are described in Supplementary data Table S1). Animals were immobilized under anaesthesia (2.5% Isoflurane in air) and positioned vertically with lead shielding designed to protect the entire body excluding the thorax. GAFchromic™ EBT-XD film (Ashland Inc., Wayne, NJ, USA) was used for the dosimetry of entrance and exit dose at each irradiation. Animals were examined for weight loss and respiratory distress daily post IR. High resolution Micro-CT imaging (Molecubes), 100 μm FDK reconstruction, was performed to examine lung fibrosis development each month from 4 months post irradiation. The 3D lung reconstruction and fibrosis classification (24) were performed using VivoQuant 2021 (VivoQuant) and ImageJ/FIJI (ImageJ) software. For the 3D lung reconstruction, connected Hounsfield Units (HU) (bottom panel of figure 5B) were detected. Air filled structures represent with HU around –600 and complete lung detection was set between –800 and –100 HU. Increased lung density was detected by the loss of connected HU between the set margins. Upon reaching the ethical endpoint, mice were anesthetized (2.5% Isoflurane in air) and underwent CT scanning prior to euthanasia by cervical dislocation. Lungs were isolated and histology was performed to detect areas of affected lung by pulmonary fibrosis. Note that mice that were still alive at day 200 (final endpoint) were euthanised.

### Single cell RNA sequencing

Single cell RNA sequencing was performed on three controls provided by Curras *et al.* (34), 1 CONV, 1 CONV AsiDNA™ and 1 FLASH female C57BL6/J mice 5 months post 13 Gy thorax irradiation. The protocol and data processing procedures were performed as previously described (34). In brief, following lung tissue dissociation, single cell samples for RNA sequencing were prepared using the droplet based scRNA-seq system (10x GENOMICS) followed by lysis of encapsulated single cells, RNA capturing, cDNA production, amplification, purification, library preparation, and sequencing. scRNA-seq data analysis was processed through the creation of a count matrix table suitable for R (4.0.5) and analysed using Seurat package (v4.0.1.).

### Histology

For histological analysis, the lungs were removed, and gently inflated in 4% paraformaldehyde (PFA) under mild vacuum pressure (25 Torr, 1 h at room temperature). Lungs were fixed for 24 h at RT, after which they were embedded in paraffin and cut into 7-mm thick slices. The preparations were stained with hematoxylin–eosin or Masson trichrome (R.A.L. Diagnostics, #361350).

### Animal irradiation and intestine analysis

Female C57BL6/J mice were irradiated and treated as previously described (29). Lower body irradiation of 10 Gy was performed using the linear accelerator described in Ruan *et al.*, including CONV dose rate of 0.1 Gy/min and FLASH dose rate of 3000 Gy/s with beam parameters described in Supplementary data Table S2. Animals were immobilized under anaesthesia in a cradle exposing the lower body. Brass shielding was used to protect the entire animal's body excluding the abdominal region. GAFchromic™ EBT-XD film was used for the dosimetry of the exit dose for each irradiation. Animals were examined for weight loss with the endpoint set at 4 days post IR. The jejunum of the small intestine was isolated using the swiss roll technique followed by intestine histology with haematoxylin and Eosin staining as previously described (35). The count of intestinal crypts was performed over a length of 3 mm for each sample and conducted twice by independent researchers.

### *In vivo* detection of EdU, Ki67 and p21

Female C57BL6/J mice received intraperitoneal AsiDNA™ injections (100 mg/kg) for 2 consecutive days, followed by a third day with intraperitoneal AsiDNA™ injection (200 mg/kg). EdU (100 mg/kg) was injected 4 h prior to euthanasia at 0, 24, 48 and 72 h post AsiDNA™ injection. The small intestine was isolated from 3 cm after the stomach, with a total length of 10 cm intestine isolated overall, using the swiss roll technique (36). Samples were fixed in 4% PFA for 36 h, embedded in parafilm, and cut into 4 μm thick slices. These slices were then deparaffinized and hydrated following a standard protocol. DAPI (0.5 μg/ml) staining and EdU detection were performed using BaseClick EdU IV Imaging kit 488 M in accordance with the manufacturer's protocol. EdU positive cells were detected using the 3D SIM Upright Widefield microscope (Leica), and quantified using a nuclear segmentation algorithm (Cellpose) and MIC-MAQ macro (supplementary Materials and methods), applied on nuclear DAPI signal and the individual EdU cell signal. Furthermore, standard immunofluorescence was conducted to detect Ki-67 (FISHER, MA5-14520, 1:200) and immunohistochemistry staining to detect p21 (Tebu-Bio, E-AB-70068, 1:200).

### Statistical analysis

All statistical analyses were performed using GraphPad Prism (v 7.03). Statistical significance was set at *P < 0.05, **P < 0.01, ***P < 0.001 and, ****P < 0.0001. All statistical information is presented in the figures and figure legends.

## Results

### AsiDNA™ induces a G1/S arrest in normal proliferating epithelial cells and primary fibroblasts.

**Figure 1.**
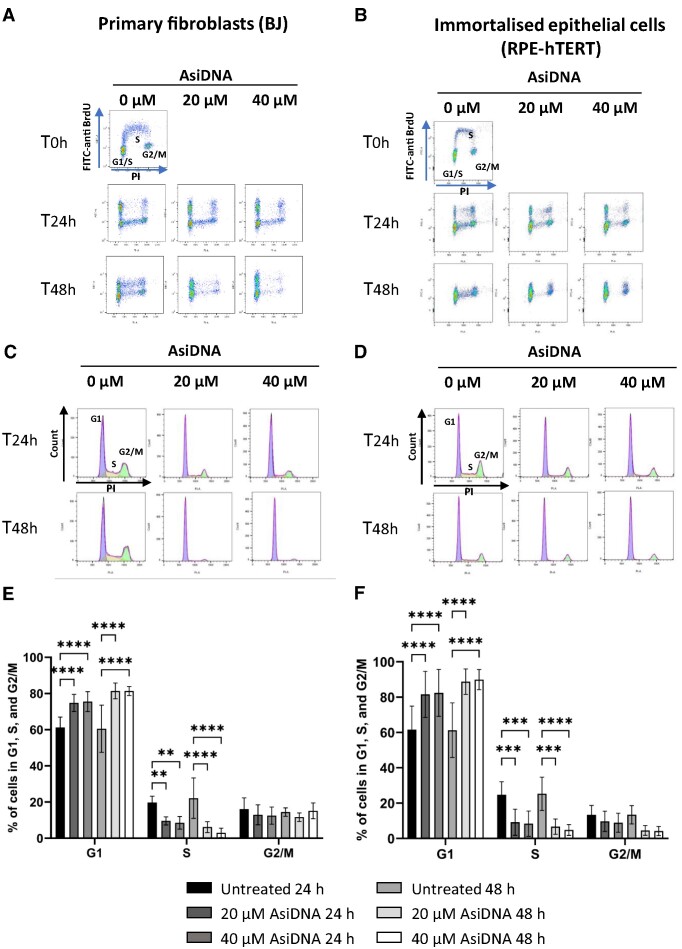
AsiDNA™ induces a G1/S arrest in healthy cells *in vitro*. Cells were pulse-labelled with BrdU following incubation with 20 and 40 μM of AsiDNA™ for 24 and 48 h. Representative images of the bivariate analysis by flow cytometry of BrdU incorporation versus DNA content (PI) in (**A**) BJ and (**B**) RPE-hTERT cells. The deconvolution of the cellular DNA content frequency histograms allows the identification of G1 phase (purple), S-phase (orange), and G2/M-phase (green) in (**C**) BJ, and (**D**) RPE-hTERT cells. The percentage of cells in G1, S, and G2/M is shown in (**E**) for BJ, and in (**F**) for RPE-hTERT cells. Data are expressed as mean ± standard deviation (*n* = 8–9) with significance given by two-way ANOVA, Tukey's multiple comparison tests, and represented above the bar plots. Statistical significance was set at * *P* value < 0.05, ** *P* value < 0.01, *** *P* value < 0.001 and **** *P* value < 0.0001.

The adverse side effects induced by radio- and chemo-therapies are derived from damage to dividing normal cells, resulting in cell death within the healthy tissue (37,38). Cell cycle arrest has previously been demonstrated to protect normal cells against cytotoxic radio- and chemo-therapies (39,40). Consequently, in the present study, we assessed cell cycle progression in a panel of normal human cells treated with AsiDNA™. For this, primary skin fibroblasts (BJ), and immortalised normal epithelial cells (RPE-hTERT) were exposed to 20 and 40 μM of AsiDNA™ for 24 and 48 h, followed by cell cycle analysis using PI-BrdU bivariate flow cytometric dot plots (Figure 1A and B). The corresponding histograms showing cell cycle analysis (Figure 1C and D) allow quantification of the number of cells in each cell cycle phase (Figure 1E and F). Analysis of these results indicated significant cell cycle arrest at the G1/S boundary, which implies an accumulation of cells in G1 and an affiliated decrease of S-phase cells (Figure 1E and F). Similar results were obtained using MRC-5 (primary lung fibroblasts) and VH10-hTERT (immortalised fibroblasts) (Supplementary Figure S1). It should be noted that AsiDNA™ treatment did not induce a G2/M arrest (absence of cell accumulation in G2/M) nor S phase arrest (absence of BrdU negative cells) following AsiDNA treatment (Figure 1 and Supplementary Figure S1).

### A functional DNA-PK/p53/p21 pathway is required to promote AsiDNA™-induced G1/S arrest in normal proliferating epithelial cells and fibroblasts

**Figure 2.**
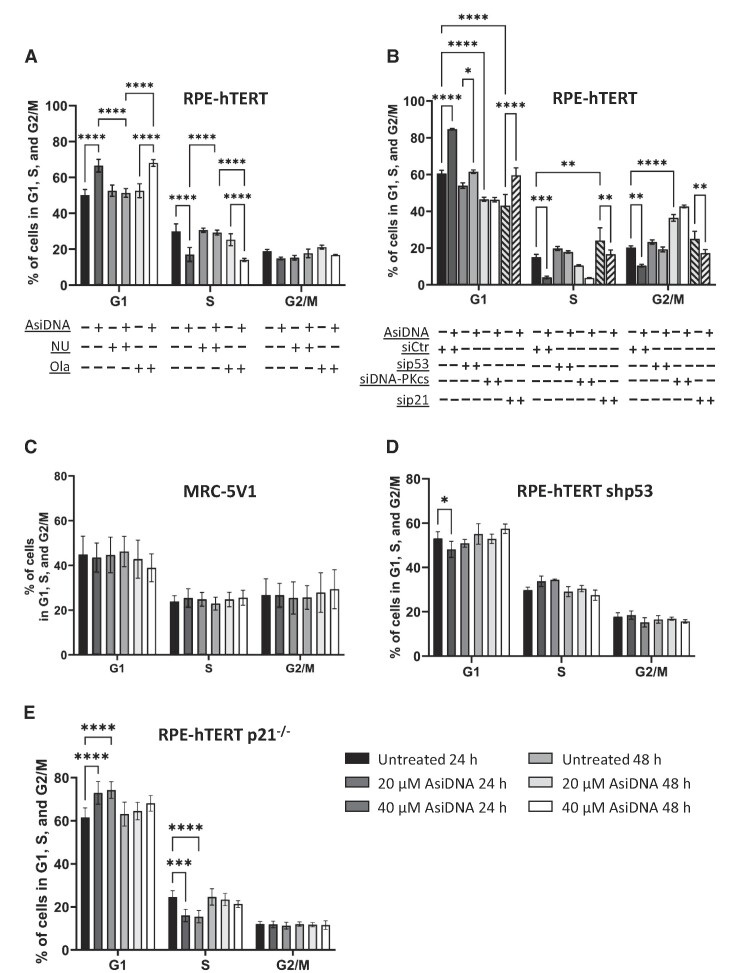
AsiDNA™-induced G1/S arrest *in vitro* is dependent on DNA-PK, p53, and p21. (**A**) RPE-hTERT cells were pre-treated with 1 μM olaparib (Ola) or 10 μM NU7026 (NU) for 1 h before addition of 20 μM AsiDNA™. The percentage of cells in G1, S and G2/M was analysed by flow cytometry 48 h post-AsiDNA™ treatment based on PI staining. (**B**) RPE-hTERT cells were transiently transfected with small inhibitory RNA (siRNA) silencing DNA-PKcs, p53, or p21 before being exposed to AsiDNA™ for 48 h. The percentage of cells in G1, S and G2/M was analysed by flow cytometry based on PI staining. (**C**) MRC-5V1, (**D**) RPE-hTERT shp53 and (**E**) RPE-hTERT p21^−/−^ cells were treated with 20 and 40 μM of AsiDNA™ for 24 and 48 h. The percentage of cells in G1, S and G2/M was analysed by flow cytometry at the end of AsiDNA™ treatment based on PI staining. All the data are expressed as mean ± standard deviation (*n* = 3–9) with significance given by two-way ANOVA, Tukey's multiple comparison tests, and represented above the bar plots. Statistical significance was set at * *P* value < 0.05, ** *P* value < 0.01, *** *P* value < 0.001 and **** *P* value < 0.0001.

Nol8, which is structurally similar to AsiDNA™ but with an 8 bp instead of 32 bp nucleotide strand, showed no capacity to activate PARP and DNA-PK (Supplementary Figure S2A and B). Furthermore, although p21 was moderately induced at 48h following Nol8 treatment, no significant G1/S arrest was observed (Supplementary Figure S2C–E). These results prompted us to investigate the role of PARP and DNA-PK in AsiDNA-induced cell cycle arrest. PARP and DNA-PKcs activity were inhibited using olaparib (41), and NU7026 (42), respectively, and cell cycle progression was examined upon combined treatment with AsiDNA™. The inhibition of DNA-PK activity, but not of PARP, was able to prevent AsiDNA™-induced G1/S arrest in RPE-hTERT and BJ (Figure 2A and Supplementary Figure S3A). This dependency on DNA-PK activation was further confirmed using cells in which the expression of DNA-PK was down-regulated (Figure 2B and Supplementary Figure S3B). In support, previous research revealed that the p53–p21 axis is an important pathway controlling G1/S arrest upon activation of the DNA damage response (43). Therefore, the contribution of this axis to AsiDNA™-induced G1/S arrest was further examined. Cell lines with inactive p53 (MRC-5V1, Figure 2C) or with downregulated p53 expression (RPE-hTERT sip53, Figure 2B and RPE-hTERT shp53, Figure 2D), displayed no AsiDNA™-induced G1/S arrest. The results obtained following the downregulation of p21 expression (Figure 2B) or p21 inhibition (Supplementary Figure S3C) in RPE-hTERT cells revealed a leaky phenotype with a minor accumulation of cells at the G1/S transition, concomitantly with a minor decrease of S-phase cells following AsiDNA™ treatment. The efficient downregulation of DNA-PKcs and p53 expression was confirmed with a partial inhibition of p21 expression using western blot analysis (Supplementary Figure S3D). To overcome this problem, RPE-hTERT cells with p21 gene knock-out were acquired (31). The result revealed a transient G1/S arrest at 24 h of treatment that is not sustained at 48 h (Figure 2E), demonstrating an essential role of p21 to establish a robust AsiDNA-induced G1/S arrest.

To further confirm the importance of the DNA-PK/p53/p21 axis to trigger AsiDNA™-induced G1/S arrest, the expression levels of p53 and p21 in response to 24h and 48h of AsiDNA™ treatment were examined by western blot in RPE-hTERT, RPE-hTERT shp53, RPE-hTERT p21^−/−^, and RPE-hTERT cells treated with NU7026. The results revealed that AsiDNA™ exposure initiated p21 induction in RPE-hTERT cells that was absent in RPE-hTERT p21^−/−^ cells (Supplementary Figure S3E), in RPE-hTERT shp53 cells (Supplementary S3F), or in RPE-hTERT treated with DNA-PKcs inhibitor NU7026 (Supplementary Figure S3G). Similarly, down-regulation of p53 expression in primary fibroblasts additionally resulted in the loss of the AsiDNA-induced G1/S arrest (Supplementary Figures S3B). Collectively, these results demonstrate that DNA-PK activity is required to promote the p53-dependent transcriptional activation of p21 which leads to the G1/S arrest induced by AsiDNA™. Furthermore, AsiDNA™-induced G1/S arrest was reversible. Following the removal of AsiDNA™ treatment, cells restarted their cell cycle and p21 expression returned to basal level (Supplementary Figure S4).

### P53-proficient tumour cells show no G1/S arrest upon AsiDNA™ treatment

**Figure 3.**
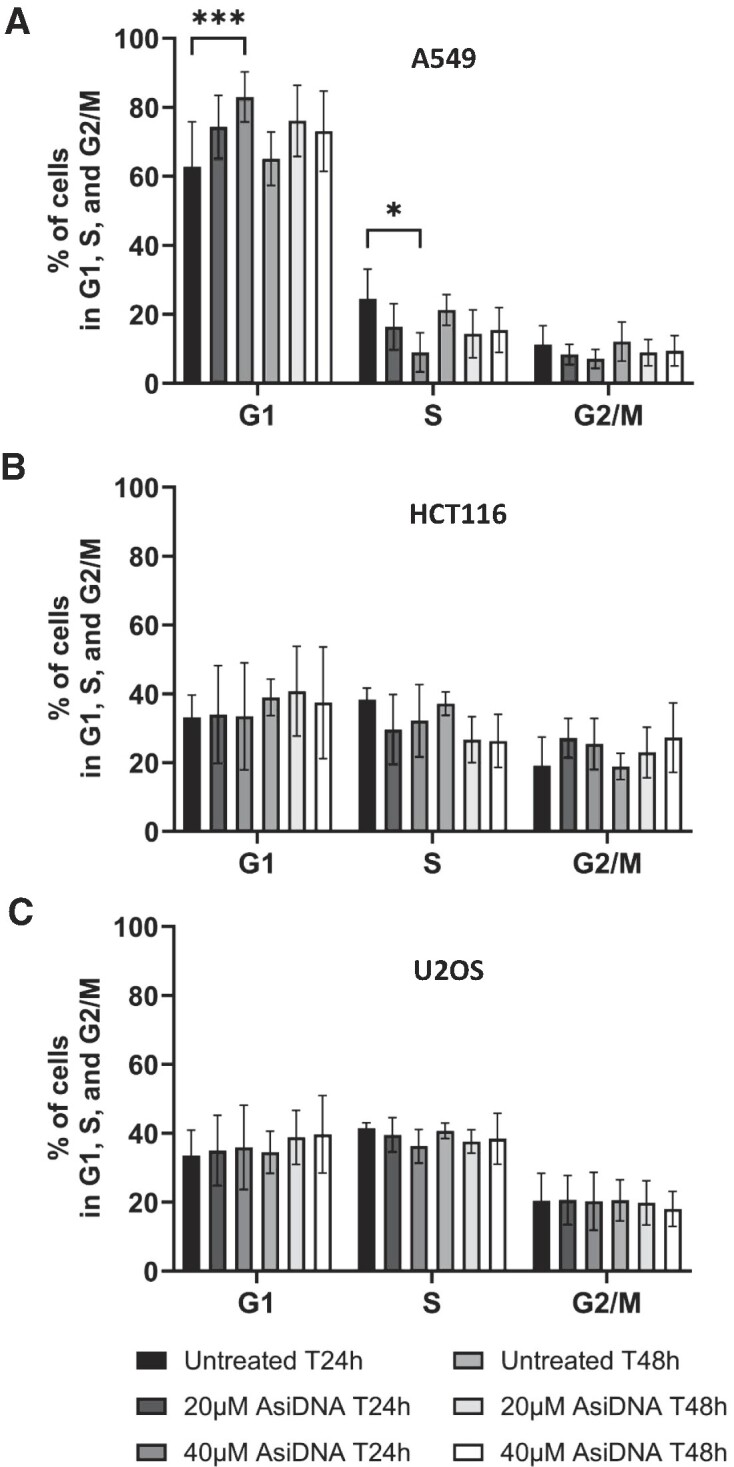
No effect of AsiDNA™ treatment on cell cycle progression in p53 proficient tumour cells *in vitro*. (**A**) A549, (**B**) HCT116 and (**C**) U2OS tumour cells were exposed to 20 and 40 μM of AsiDNA™ for 24 and 48 h. The percentage of cells in G1, S and G2/M was analysed by flow cytometry at the completion of AsiDNA™ treatment, based on PI staining. Data are expressed as mean ± standard deviation (*n* = 5–7) with significance given by two-way ANOVA, Tukey's multiple comparison tests, and represented above the bar plots. Statistical significance was set at *** *P* value < 0.001.

Standalone AsiDNA™ treatment has previously been demonstrated to cause toxicity in malignant cells, irrespective of their p53 status, and exerted no toxicity against normal cells (7,8,18,44). As such, we subsequently investigated AsiDNA™-induced cell cycle arrest in p53 proficient tumour cells (A549, HCT116 and U2OS). Among the three p53 proficient tumour cell lines, U2OS and HCT116 displayed no G1/S arrest after AsiDNA™ treatment. In A549 cells, a transient increase in G1 phase concomitantly with a transient decrease in S-phase was observed at 24 h that was absent at 48 h following AsiDNA™ treatment (Figure 3). Despite a functional p53, the lack of G1/S arrest was further confirmed by the absence of p21 induction upon AsiDNA™ treatment in the examined tumour cell lines (Supplementary Figure S5A). As expected, no G1/S arrest was observed following AsiDNA™ treatment in DAOY, a p53-deficient tumour cell line (Supplementary Figure S5B). Collectively, these results strongly suggested that AsiDNA-induced p53-dependent G1/S arrest is restricted to proliferating normal epithelial cells and fibroblasts. As previously observed following the impact of AsiDNA treatment on normal cell cycle progression, AsiDNA™ identically did not activate the G2/M checkpoint (Figure 3 and Supplementary Figure S5B) and the intra S-phase checkpoint (data not shown) in the examined tumour cell lines.

### AsiDNA™-induced G1/S arrest promotes cell survival in response to ionizing radiation in normal epithelial cells and fibroblasts

**Figure 4.**
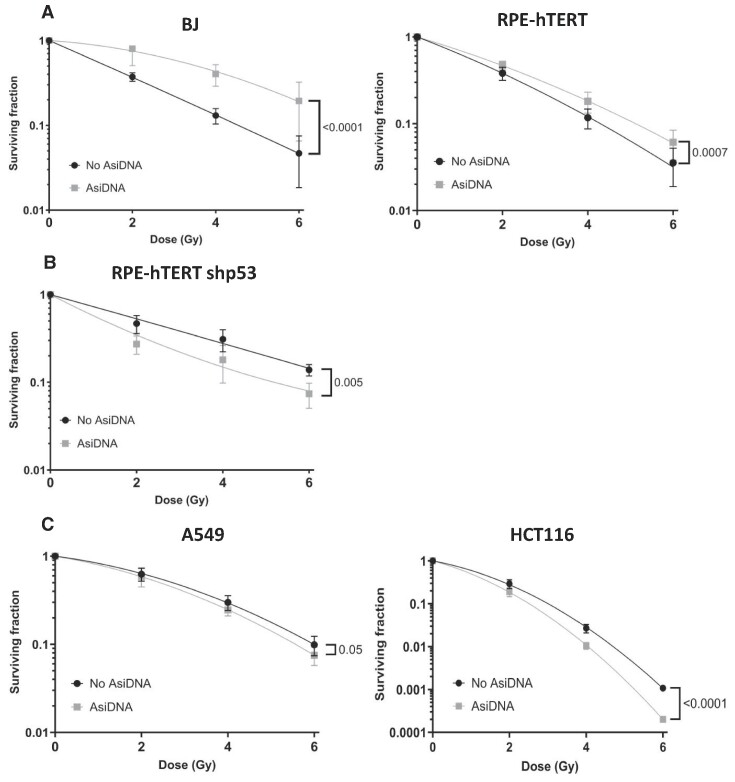
AsiDNA™ treatment protects p53-proficient normal cells, but not p53-proficient tumour cells, from radiation-induced toxicity *in vitro*. (**A**) p53 proficient normal cells (BJ and RPE-hTERT), (**B**) p53 deficient normal cells (RPE-hTERT shp53) and (**C**) p53 proficient tumour (HCT116 and A549) cells were pre-treated with AsiDNA™ 24 h before being co-exposed to increased doses of ionizing radiation (0–6 Gy). The survival fraction was determined 8–12 days post-treatment using a clonogenic survival assay. Data are expressed as mean ± standard deviation (*n* = 3 for BJ, RPE-hTERT shp53, HCT116 and A549; *n* = 4 for RPE-hTERT), fitted to the linear-quadratic model as a function of dose with significance given by nonlinear fit using GraphPad Prism.

The G1/S cell cycle checkpoint primarily prevents damaged DNA from being replicated during the S phase, which can be either mutagenic or lethal for the cells. It is therefore hypothesized that AsiDNA™-dependent G1/S arrest could protect healthy cells from radiation-induced toxicity. To test this hypothesis, normal human cell lines (BJ and RPE-hTERT) were treated with AsiDNA™ prior to irradiation. An increase in cell survival was observed in p53 proficient normal cells treated with AsiDNA compared to cells without AsiDNA™ treatment (Figure 4A). Importantly, there was no protection from radiation induced toxicity in cells lacking the AsiDNA™-induced G1/S arrest as observed in RPE-hTERT shp53 cells (Figure 4B) and p53 proficient tumour cells (A549 and HCT116) (Figure 4C). It is worth noting that the extent of radioprotection or radiosensitivity is cell line dependent. Indeed, we observed that primary BJ fibroblasts displayed a higher radioprotection by AsiDNA (SF_6Gy/-AsiDNA_ ≈ 0.05, SF_6Gy/+AsiDNA_ ≈ 0.2) compared to immortalized RPE-hTERT cells (SF_6Gy/-AsiDNA_ ≈ 0.035, SF_6Gy/+AsiDNA_ ≈ 0.06). Similarly, HCT116 cells displayed a higher radiosensitivity conferred by AsiDNA (SF_6Gy/-AsiDNA_ ≈ 0.001, SF_6Gy/+AsiDNA_ ≈ 0.0002) in comparison to the radioresistant A549 tumour cells (SF_6Gy/-AsiDNA_ ≈ 0.1; SF_6Gy/+AsiDNA_ ≈ 0.07). These *in vitro* results demonstrate the essential role of the AsiDNA™-induced G1/S arrest in protecting explicitly normal cells, with a functional p53, against radiation-induced toxicity.

### AsiDNA™ alleviates radiation-induced lung fibrosis in mice

**Figure 5.**
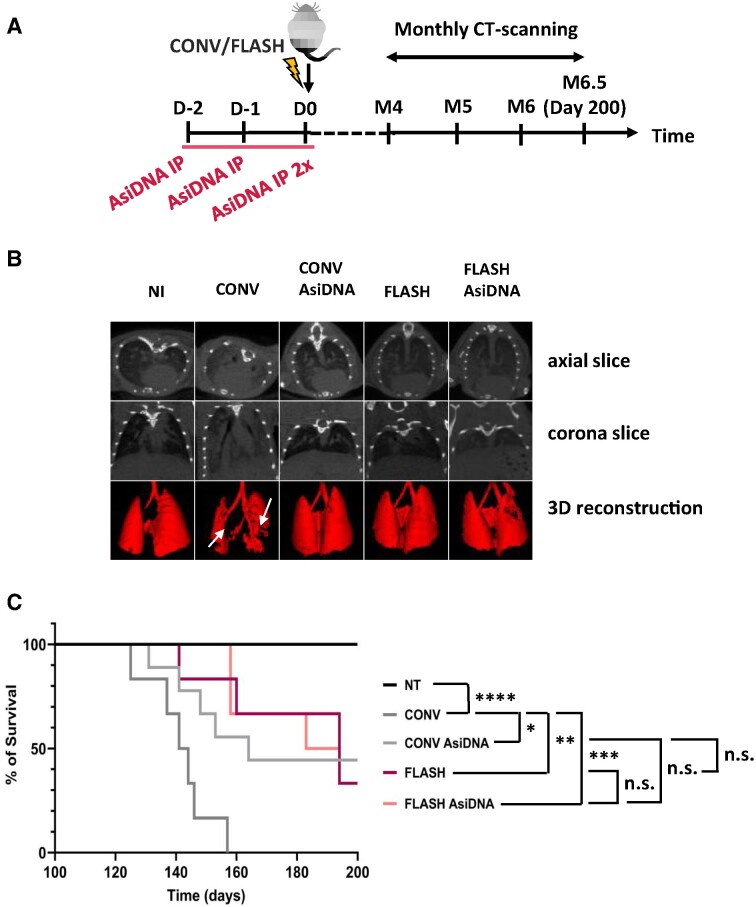
AsiDNA™ delayed the onset of radiation-induced pulmonary fibrosis *in vivo*. (**A**) Scheme of the experimental protocol. C57BL6/J mice were treated for 3 consecutive days with AsiDNA™ followed by 13 Gy CONV or FLASH irradiation of the thorax on the final day. Retro-orbital blood sampling was performed from 1 week up to 5 months post-treatment. CT scans were recorded from 4 to 6.5 months (sacrifice) post-treatment, and lobes of the lung were collected at the day of sacrifice (day 200) for histologic analysis. (**B**) Representative CT scans of lung from untreated mice (NI) or 5 months post-irradiation at 13 Gy CONV or FLASH radiotherapy alone or combined with AsiDNA™. Images are obtained using micro-CT imaging, high resolution, and 100 μm reconstruction by Molecubes software (Molecubes, Belgium). Representative images are shown with the CT axial slice (top), CT coronal slice (middle) and 3D lung reconstruction of connected Hounsfield Units −800 to −100 (bottom). Images were obtained using VivoQuant software (Konica Minolta Company, Japan). (**C**) Kaplan–Meier representation of animal surviving fraction displayed in days post-treatment. Data are expressed with significance given by survival, curve comparison, and Logrank test. Significance: not significant, ns; * *P*< 0.05; ** *P*< 0.01, *** *P*< 0.001, **** *P*< 0.0001. The statistical analysis gave NT versus CONV is < 0.0001, NT versus CONV AsiDNA is 0.003, NT versus FLASH 0.002, NT versus FLASH AsiDNA is 0.0015, CONV versus CONV AsiDNA 0.0178, CONV versus FLASH 0.0053 and FLASH versus FLASH AsiDNA is not significant.

To demonstrate that AsiDNA™ is similarly able to protect normal tissue against radiation-induced toxicity *in vivo*, radiation-induced lung fibrosis in C57BL/6J mice, a well-established model of late-responding radiation toxicity, was used (45). As FLASH-RT alleviates radiation-induced lung fibrosis in mice (23,24), we took advantage of hosting an electron accelerator that can perform radiotherapy modalities in both CONV-RT and FLASH-RT mode, enabling the examination of a possible gain of normal tissue protection following combined AsiDNA™ and FLASH-RT treatment. Mice were divided into five groups (*n* = 6–9), sham-irradiated, exposed to 13 Gy CONV ± AsiDNA™ or 13 Gy FLASH ± AsiDNA™, through bilateral thorax irradiation. Lung fibrosis was evaluated using computed tomography (CT) from 4 months post-irradiation (Figure 5A). Each lobe of the lung was collected for histopathological analysis and single cell RNA sequencing, directly following euthanasia.

CT scans taken 5 months post irradiation revealed increased levels of fibrosis in the CONV-RT treated group, while fibrosis was absent in the mock-treated group and much less pronounced in the combined CONV-RT with AsiDNA™ treated group (Figure 5B). A strong reduction of fibrosis was also observed in the FLASH-RT treated group, which agrees with the previously reported results (23,24), and following combined treatment with FLASH-RT and AsiDNA™ (Figure 5B). Long-term follow-up of survival post irradiation demonstrated that AsiDNA™ delayed or even reduced the onset of lethal lung fibrosis when combined with CONV-RT (Figure 5C). Similar results were obtained with FLASH-RT, as previously reported (23), and with the combined treatment of FLASH-RT and AsiDNA™ (Figure 5C). It is to note that overall survival is similar between combined CONV-RT and AsiDNA™ treatment, FLASH-RT standalone, and combined FLASH-RT and AsiDNA™ treatment. These results suggest that, at the cellular level, the effect of AsiDNA™ combined with CONV-RT could mimic the effect of FLASH-RT on reduced normal tissue toxicities. Histopathological analyses of the lobes collected at the day of euthanasia, either by reaching the ethical endpoint or the final endpoint (day 200), confirmed the onset of lung fibrosis in all groups but in the non-irradiated controls (Supplementary Figure S6, and Supplementary data Table S3).

### A decreased myofibroblast gene profile is observed in fibroblasts upon AsiDNA™ treatment

**Figure 6.**
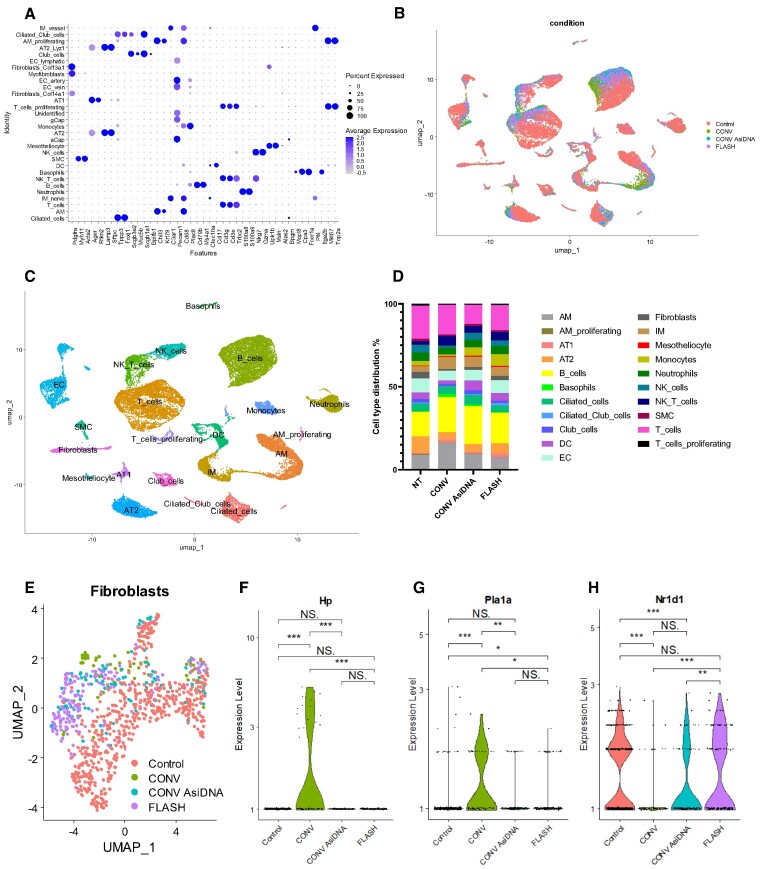
Single-cell RNA sequencing after irradiation and AsiDNA™ treatment. Identification of cell populations represented by (**A**) Dot plot of marker expression utilized for cell population identification. (**B**) UMAP visualizing the identified cell clusters separating representation of the Control (red), CONV (green), AsiDNA™ + CONV (blue), and FLASH (purple) treated samples. (**C**) UMAP visualizing the identified cell types in all samples. The individual dots signify single cells. Additionally, the created clusters are established on transcriptome resemblances. (**D**) Cell population proportions after Control, CONV, AsiDNA™ + CONV and FLASH treatment. Fibroblast populations represented by (**E**) UMAP visualizing the identified fibroblast cluster separating representation of the Control (red), CONV (green), AsiDNA™ CONV (blue) and FLASH (purple) treated samples. Pro-fibrotic markers were examined using Violin plots with myofibroblast signature genes Hp (**F**) and Pla1a (**G**), and healthy collagen homeostasis Nr1d1 (**H**). CONV irradiation upregulates the expression of Hp and Pla1a and decreases the expression of Nr1d1 compared to FLASH, CONV + AsiDNA™, and NI control, significance given by Wilcox test. (NS, *P*-value > 0.05; *, *P*-value < 0.05; **, *P*-value < 0.01; ***, *P*-value < 0.001; ****, *P*-value < 0.0001).

To characterize the similarity in cellular changes and expression signatures in lungs 5 months post CONV-RT, FLASH-RT, AsiDNA™ + CONV-RT or non-irradiated (Control) treatment, single cell suspensions were created and analysed using scRNA sequencing. By exploiting previously published single-cell datasets and known identifying markers (34), the identity of the various cell clusters was determined (Figure 6A). This analysis detected 21 distinct clusters: alveolar macrophages (AM), proliferating AM, AT1, AT2, B-cells, basophils, ciliated cells, ciliated club cells, club cells, dendritic cells (DC), endothelial cells (EC), fibroblasts, interstitial macrophages (IM), mesitheliocytes, monocytes, neutrophils, natural killer cells (NK cells), NK-T-cells, smooth muscle cells (SMC), T-cells and proliferating T-cells (Figure 6B–D). All cell types were identified independently of the received treatment modality but a radiation-induced cell proportion shift was detected in the AT2, B-cells, IM and NK-T cells (Figure 6D). Additionally, the AM, DC, Monocytes and NK-cells populations displayed altered cell proportions following CONV-RT compared to the other conditions (figure 6D). Recent findings by Curras *et al.* have identified a unique fibroblast subcluster exclusively present in irradiated mice lungs (34). In response to irradiation, fibroblasts can transition into myofibroblasts which are known to secrete and modify the extracellular matrix (ECM), including altering the collagen production, which in turn contributes to pulmonary fibrosis formation (46). To identify the contribution of fibroblasts in the development of, or the lack of, pulmonary fibrosis formation after CONV-RT, CONV AsiDNA™ treatment and FLASH-RT, myofibroblast markers expression, collagen homeostasis, fibroblast activation and EMC remodelling markers were examined within the fibroblast cell cluster. Clustering of the fibroblast resulted in the detection of a total of 891 cells, divided over the different treatment conditions (Figure 6E). Myofibroblast markers Hp and Pla1a, previously identified in Curras *et al.* (34), revealed to be substantially increased after CONV-RT standalone (Figure 6F–G). Remarkably, Nr1d1 gene expression, linked to healthy collagen homeostasis (47), was found to be significantly decreased in CONV-RT compared to the control, CONV AsiDNA™ or FLASH-RT (Figure 6H).

These results reveal an increase in fibroblast activation and myofibroblast transition together with an impact on the ECM, including collagen homeostasis, in the fibroblast cell cluster following CONV-RT standalone. This observed altered gene expression in CONV-RT exposed fibroblast was decreased or absent in fibroblasts exposed to CONV AsiDNA™ or FLASH-RT, and supports the increased pathway activation known to play an essential role in the development of pulmonary fibrosis.

### AsiDNA™ induces a cell cycle arrest in ex vivo precision cut lung slices involving DNA-PK and p53

**Figure 7.**
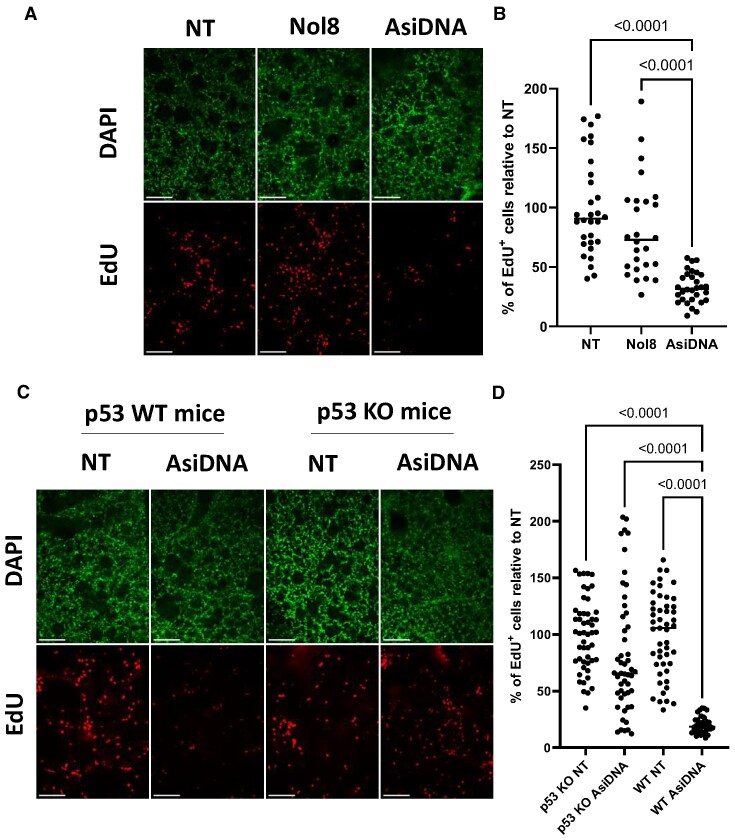
AsiDNA™ induces a DNA-PK and p53-dependent cell cycle arrest in the ex vivo model of precision cut lung slices. Precision cut lung slices (PCLS) were derived from C57BL6/J WT or p53 knock-out mice and treated with EdU upon 24 h of AsiDNA™ or Nol8 treatment. (**A**) Representative images of PCLS derived from WT mice (*N* = 2) with DAPI and EdU detection after AsiDNA™ or Nol8 treatment. (**B**) EdU positive cells detected after AsiDNA™ or Nol8 treatment (*N* = 2). EdU was detected in 6 slices per condition with 5 readouts per slice. (**C**) Representative Images of PCLS derived from WT (*N* = 2) and p53 knock-out mice (*N* = 2) with DAPI and EdU detection after AsiDNA™ treatment. (**D**) EdU positive cells detected after AsiDNA™ or Nol8 treatment of two WT mice. EdU was detected in 8–10 slices per condition with 5 readouts per slice. Data are expressed as mean ± standard deviation with significance given by two-way ANOVA, Tukey's multiple comparison test and represented above the bar plots. Scale bar = 100 μm.

To investigate the AsiDNA™-induced cell cycle arrest in the lung, *ex vivo* precision cut lung slices (PCLS) were used. Untreated C57BL6/J mice were sacrificed, and agarose inflated lungs were isolated and cut. The PCLS were treated for 24 h with AsiDNA™ or Nol8, and EdU (a marker of replicative cell division) was co-incubated for an additional 24 h. Cell nuclei were then stained for EdU incorporation (Figure 7A). A significant loss of EdU positive cells was observed following AsiDNA™ treatment compared to both untreated and Nol8 treated PCLS (Figure 7B), suggesting that DNA-PK activation by AsiDNA™ similarly triggered cell cycle arrest, likely at the G1/S border, in PCLS. To further support this conclusion, PCLS were derived from wild-type C57BL6/J mice and p53 knock-out (p53−/−) mice. Upon AsiDNA™ treatment in wild-type (WT) mice, a significant decrease in EdU positive cells was detected compared to the untreated conditions (Figures 7C and D). Additionally, the incorporated EdU positive cells of untreated PCLS in p53 WT and in p53 KO were similar. Strikingly, p53 KO PCLS treated with AsiDNA™ resulted in no significant decrease in the EdU incorporation (Figure 7D). Collectively, these results strongly support the activation of G1/S arrest induced by AsiDNA™ treatment in PCLS requiring the activation of DNA-PK and p53.

### AsiDNA™ alleviates radiation-induced intestine toxicity in mice

**Figure 8.**
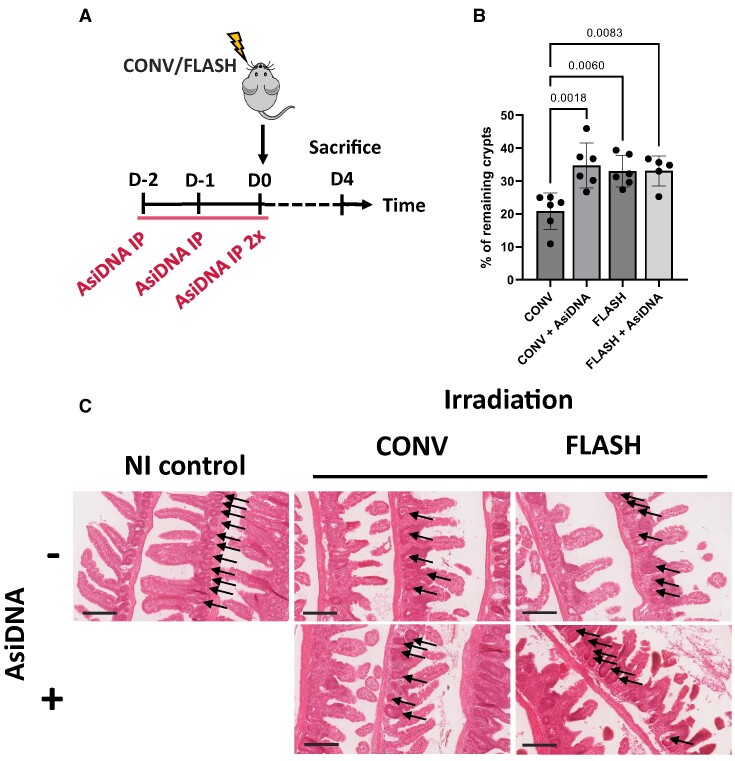
AsiDNA™ protects intestinal crypts from radiation toxicity. (**A**) Scheme of the experimental treatment timeline. C57BL6/J mice were treated for 3 consecutive days with AsiDNA™ followed by 10 Gy CONV or FLASH irradiation of the abdomen on the final day. (**B**) Small intestinal crypt survival of C57BL6/J mice 4 days after abdominal radiation, normalised to non-irradiated control mice. Data are expressed as mean ± standard deviation (*n* = 5–6) with significance given by one-way ANOVA, Tukey's multiple comparison test and represented above the bar plots. (**C**) Representative images of intestinal rolls stained with H&E from each treatment group. Arrows point to the intestinal crypts. Scale bar = 200 μm.

To study the capacity of AsiDNA™ to protect normal tissue from early responding radiation toxicity, a model of acute intestinal toxicity after whole abdominal irradiation in mice was used (29). The possible gain-of-protection by AsiDNA™ was examined in combination with CONV-RT and FLASH-RT (Figure 8A). For crypts analysis, the intestine was isolated at 4 days post treatment, and the jejunum was further processed for histochemistry analyses (Figure 8B). The number of damaged crypts in each condition was normalized to the number of crypts present in the non-irradiated mice. More crypts remained after CONV-RT combined with AsiDNA™ treatment compared with CONV-RT standalone (*P* < 0.0018) (Figure 8C). Similarly, FLASH-RT resulted in less toxicity, preserving more of the intestinal crypts than CONV-RT (*P* < 0.006). Additionally, there was no difference detected in the percentage of remaining crypts between FLASH-RT standalone and FLASH-RT combined with AsiDNA™ treatment (Figure 8C). Collectively, these results showed a gain-of-protection for AsiDNA™ only when combined with CONV-RT.

### AsiDNA™ induces a reversible cell cycle arrest *in vivo*

**Figure 9.**
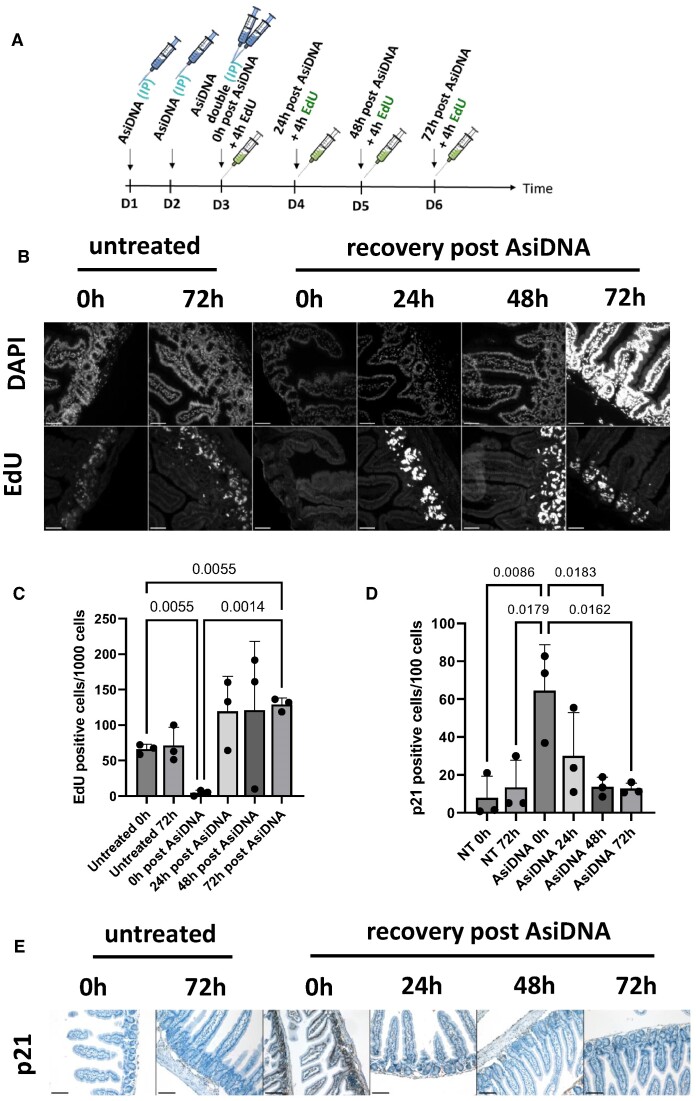
AsiDNA™-induced cell cycle arrest is reversible upon release of AsiDNA™ in intestinal normal tissue. (**A**) Scheme of the experimental treatment timeline. C57BL6/J mice were treated for 3 consecutive days with AsiDNA™ followed by 0–72 h of recovery. Thereafter, mice received EdU for 4 h prior to sacrifice. (**B**) Representative images of intestinal rolls stained with DAPI and Click-iT^TM^ EdU AlexaFluor^TM^ 488 for each treatment group. (**C**) EdU positive cells per 1000 detected cells in small intestinal crypts after AsiDNA™ treatment. A total number of 14 000–16 000 cells per mouse were scored. Data are expressed as mean ± standard deviation (*n* = 3) with significance given by one-way ANOVA, Brown-Forsythe and Welch tests. Scale bar = 50 μm. (**D**) p21 positive cells per 100 detected cells in small intestinal crypts after AsiDNA™ treatment. A total number of 6000–11 000 cells per mouse were scored. Data are expressed as mean ± standard deviation (*n* = 3) with significance given by one-way ANOVA, Tukey's multiple comparison test and represented above the bar plots. (**E**) Representative images of intestinal rolls stained by immunohistochemistry to detect p21 expression in each treatment group. Scale bar = 200 μm.

To demonstrate the capacity of AsiDNA™ treatment to arrest normal cell division *in vivo*, we used a well-established intestine model which exhibits a high rate of cell proliferation within the small intestinal crypts (48,49). EdU incorporation in intestine crypt cells of C57BL6/J mice was examined at 0, 24, 48 and 72h post AsiDNA™ treatment, and the small intestine was isolated for immunohistochemistry analyses 4 h after EdU incorporation (Figure 9A). A significant loss of EdU positive cells without any decrease in Ki67 was observed immediately following the final AsiDNA™ injection, compared to the untreated group at time 0 h or 72 h (Figures 9B and C, Supplementary Figure S7). However, this reduction was only transient, as the level of EdU positive cells has recovered at 24, 48 and 72 h post AsiDNA™ treatment. Strikingly, this level exceeds that of the control groups suggesting a boost of cell proliferation upon release from AsiDNA™ (Figure 9C). As the reported *in vitro* G1/S arrest relies on p21 induction, p21 initiation in the small intestinal crypts in response to AsiDNA™ treatment was monitored. The number of p21 positive (p21^+^) cells was reduced in untreated groups (average of 8 and 13 p21^+^ cells per 100 cells at 0h and 72h, respectively) but significantly increased upon AsiDNA™ treatment (average of 64 p21^+^ cells per 100 cells, 0h post AsiDNA™) (Figures 9D and E). Most importantly, the number of p21 positive cells decreased rapidly at 24h post treatment (average of 20 p21^+^ cells per 100 cells, 24 h post AsiDNA™) reaching a basal level at 48 and 72 h post treatment (average of 13 p21^+^ cells per 100 cells, 48 and 72 h post AsiDNA™) (Figure 9D). Taken together, these results demonstrated that the loss of DNA replication following AsiDNA™ treatment, as revealed by the decline of EdU incorporation, correlates with p21 induction, while recovery of EdU incorporation post-treatment correlates with a decrease in p21 initiation.

## Discussion

The capacity of radiotherapy to damage and eradicate tumour cells comes at the expense of toxicity to the normal tissue, causing severe patient distress and leads to critical conditions in the treatment delivery. One approach to reduce or mitigate these toxic side-effects is to utilise chemical or biological agents as radioprotectors, administered in parallel to radiotherapy delivery (50). The ideal radioprotector exhibits low toxicity and exclusive protection of normal cells against the harmful effects of radiation, without compromising the cytotoxic effects on cancer cells. In recent years, our laboratory has developed a new class of drugs mimicking DNA DSBs that can disrupt the DNA repair machinery of cancer cells, thereby enhancing the antitumoral action of radiation (9,51). The leading molecule used in pre-clinical and clinical studies, termed AsiDNA™, is well tolerated, does not induce normal tissue toxicity, and allows increased treatment duration, (17,19–21) all indicating its suitability as a radioprotector. AsiDNA™ was designed based on its ability to bind and activate PARP and DNA-PK, with the aim of destabilizing the DNA repair machinery (9). Although activation of DNA-PK occurs in tumours as well as in normal cells, only tumour cells are sensitive to AsiDNA™ treatment (7,52).

The G1/S cell cycle checkpoint is responsible for ensuring that the optimum conditions are reached for a cell to undergo successful cell division, through the sensing of both mitogens and DNA damage (53). One of the key players of this checkpoint is the transcription factor p53 (54). p53 transactivates numerous target genes involved in the induction of the cell cycle arrest and/or apoptosis (55). In the present study, we demonstrated that in p53 proficient normal cells, AsiDNA™ treatment results in p53 activation, leading to p21 induction which, in turn, initiates a reversible G1/S cell cycle arrest. Normal cells deficient in either DNA-PK, p53, or p21 are unable to arrest at the G1/S boundary following AsiDNA™ treatment. Pull-down experiments with biotinylated AsiDNA™ have revealed that DNA-PK binds to AsiDNA™ in cellulo (M. Dutreix, unpublished results). Several studies have shown that structured DNA, single-stranded DNA, and damaged DNA promote the interaction of DNA-PK with p53 (56–58). We propose that AsiDNA™ can serve as a platform to connect DNA-PK and p53, resulting in p53 activation. In line with this assumption, our results revealed that MRC-5 primary cells can arrest at the G1/S boundary in response to AsiDNA™ treatment, while MRC-5V1 cells failed to do so. MRC-5V1 are SV40-transformed cells instigating p53 protein blockage by the SV40 large T antigen (59), which abrogates the DNA binding activity and transcriptional activity of p53. These results agree with a previous report showing the absence of p21 induction and G1/S arrest in MRC-5V1, that were present in MRC-5 primary cells, in response to ionizing radiation (60).

The p53 proficient tumour cell lines used within this study (A549, U-2 OS and HCT116) did not arrest at the G1/S boundary upon AsiDNA™ treatment, correlated with a lack of p21 induction. However, a p53- and p21-dependent G1/S arrest in tumour cells has been observed in response to DNA damaging agents such as chemical compounds (61–63) and ionizing radiation (64–66). Regardless, radiation-induced G1/S arrest is ATM/Chk2/p53/p21-dependent (67) whereas AsiDNA™-induced G1/S arrest, identified within this study, revealed its dependency on DNA-PK/p53/p21. Considering the two types of response, not only the DNA substrate differs, one being genomic DNA the other a synthetic DNA fragment, but most importantly, ATM has not been found associated to AsiDNA™ in pull-down experiments (Marie Dutreix, unpublished data), unlike DNA-PK. This also highlighted by the fact that immunofluorescence experiments failed to detect ATM phosphorylation in the nucleus upon AsiDNA treatment (9). As outlined in (68), in order for p53 to accumulate in cells and to transactivate target genes, the degradation of p53 must be inhibited, the p53 protein must accumulate in the nucleus and the sequence-specific binding activity must be induced. We have previously shown that DNA-PK is recruited, and consequently activated in response to AsiDNA™ in all normal and tumour cell lines examined so far, as revealed by phosphorylation of H2AX ((7,9), and unpublished data). These results suggest that either the recruitment of p53 to DNA-PK/AsiDNA™ complex, and downstream transactivation of p21, is impaired in p53 proficient tumour cells, unlike p53 proficient normal cells or p53 is recruited but cannot exert properly its transcriptional activity. Normal and cancer cells differ by several phenotypic and genotypic modifications very well documented in ((69) and references therein). Among them, it is well describing that the metabolism of cancer cells differs from that of normal cells (70). We have previously demonstrated that PARP is another important protein that is activated by AsiDNA™ (9). High PARP activity leads to energy exhaustion in part due to NAD depletion (71). Notably, p53 is not only a key metabolic regulator, including NAD metabolism (72), but there is a cross-talk between p53, NAD homeostasis and PARP (73). Fischbach *et al.* revealed that p53 binds non-covalently to PARylated PARP-1, which in turn PARylates p53 (74). In another study, Wang *et al.* reported that the rapid recruitment of p53 (within seconds), to laser-induced sites of DNA damage, depends upon PARylation of p53 and closely mirrored the recruitment of PARP and Ku70 to the sites of damage (75). However, this rapid accumulation of p53 did not correlate with the presence of transcriptional activity. Most importantly, PARP inhibition delayed the recruitment of p53 but did not suppress the eventual recruitment of p53 at the sites of damage (75). Our research revealed that PARP and DNA-PK are activated upon binding to AsiDNA™ in normal and tumour cells, assessed by PARylation and phosphorylation of H2AX, respectively (this study, see also (9)). However, the inhibition of PARP by olaparib was unable to prevent AsiDNA™-induced G1/S arrest suggesting that PARP is not a key mediator in triggering p53 transcriptional activity. Taken together, we hypothesize that the lack of p53 transcriptional activity in AsiDNA-treated p53 proficient tumour cells is likely due to the inability of p53 to connect to the AsiDNA™/DNA-PK complexes. A deeper understanding of the subcellular localization and mobility of p53 and AsiDNA™ in tumour and normal cells is required to validate or rule out our hypothesis.

In the present study, we confirmed the presence of the activated G1/S checkpoint in complex *ex vivo* and *in vivo* biological models. The *ex vivo* PCLS model retains comparable viability and tissue homeostasis during a cultivation period of 1 to 3 days (76), and can be used to monitor cell proliferation using EdU incorporation (32). AsiDNA™-treated PCLS derived from p53 WT mice revealed a severe decrease of EdU positive cells, while this decrease was absent in PCLS derived from p53 knock-out mice, or in PCLS p53 WT treated with Nol8, an AsiDNA™-like molecule unable to activate DNA-PK (5). This provides further evidence that AsiDNA™ treatment in PCLS results in DNA-PK/p53-dependent G1/S arrest. Additional conformation was observed *in vivo* where the capacity of AsiDNA™ to induce the G1/S arrest in the intestine was demonstrated with a severe decrease of EdU incorporation in the intestinal crypts of mice directly after AsiDNA™ treatment. It furthermore disclosed a full recovery of crypt division 24–48 h post-AsiDNA™ treatment, verifying the reversibility of the G1/S arrest. Remarkably, the decrease of EdU incorporation immediately after AsiDNA™ and its recovery post treatment, were both concomitantly associated with an increase of p21 expression, followed by its decrease. This provides further evidence that AsiDNA™ can activate a reversible G1/S checkpoint in PCLS. Moreover, the recovery of cell division in the crypts is associated to an excess in EdU-positive dividing cells from 24 h post AsiDNA™ treatment. This boost in normal cell proliferation post drug treatment is a phenomenon that has been previously identified (77,78). As AsiDNA™ has widely been identified to not result in toxicity, this compensation occurrence might accompany the contribution to improved tissue recovery.

In response to DNA damaging agents, dividing cells stall or arrest their cell cycle progression to detect and repair DNA damage before they can resume the cell cycle (67). This contributes to the maintenance of both genome integrity, and overall survival. The results within this research revealed a significant increase of the survival of normal cells, *in vitro*, upon radiotherapy combined with AsiDNA™, compared to standalone treatment. This radioprotection was absent in tumour cells, independent of the p53 status, as well as in normal cells with a p53 deficient status. This confirms the necessity of an active and intact DNA-PK/p53/p21 cascade to exploit the radioprotection of normal tissue driven by AsiDNA™-induced G1/S arrest. More interestingly, this arrest overcomes the radiosensitizing activity of AsiDNA™ for which this molecule has been designed for (51).

The protective capacities of AsiDNA™ were similarly identified *in vivo* in the intestine crypt survival, as early model of radiation induced toxicity (29), and in the radiation-induced lung fibrosis, as late model of radiation induced toxicity (23). Combined CONV-RT with AsiDNA™ treatment resulted in an increase in crypt survival, compared to CONV-RT standalone, confirming the capacity of AsiDNA™ to protect against radiation induced toxicity *in vivo*. The intestinal epithelium regenerates itself through the proliferation and differentiation of stem cells (79). The intestine can therefore fully regenerate from any type of damage if the stem cells remain functional, revealing the capacity of AsiDNA™ to protect the stem cells in the crypts by acting at the G1/S transition. Remarkably, pharmacologic inhibition of the G1/S transition by CDK4/6 inhibitors prior to radiation (80), or by UCN-01 prior to chemotherapy (40) also protect the gastrointestinal epithelium in mice. The protective capacities of AsiDNA™ treatment were confirmed *in vivo*, with similar results obtained on the late radiotoxicity model of radiation-induced pulmonary fibrosis. Here, once again, AsiDNA™ combined with CONV-RT revealed increased protection of the lung to radiation toxicity presented by a delay in the onset of radiation induced fibrosis, compared to CONV-RT standalone.

Finally, the combination of AsiDNA™ with FLASH-RT, a RT modality that has been shown to alleviate radiation-induced toxicity (23,29), was explored. In one respect, FLASH-RT was shown to be less toxic, with decreased early (intestine model) and late (lung model) toxicity compared with CONV-RT, thereby reconfirming the FLASH effect. However, AsiDNA™ combined with CONV-RT did not result in the same delay in the onset of fibrosis compared to FLASH radiotherapy standalone, while combined AsiDNA™ CONV-RT treatment was as efficient as FLASH-RT standalone at protecting intestinal crypts. This may be explained by the possible limitations in the capacity of AsiDNA™ to interfere with the complex, and still relatively unknown, mechanism driving fibrosis in late responding tissues (81). Moreover, AsiDNA™ combined with FLASH-RT treatment did not result in any additive effect on the protection of toxicity in the intestinal crypts nor the lung, compared to FLASH-RT as standalone treatment. Interestingly, single cell RNA sequencing of irradiated lungs revealed a closer resemblance between CONV AsiDNA™ and FLASH-RT in profibrotic gene signatures within the fibroblast population, in comparison to CONV-RT standalone. Similar results on profibrotic gene signatures were observed in the alveolar macrophages population (A. Sesink, and P.-M. Girard, unpublished results). Fouillade et al. (2020) revealed that FLASH irradiation tends to minimize DNA damage, compared to CONV irradiation, resulting in less induction of senescent cells and less proliferation of stem/progenitor cells to replace the damage tissue. The authors suggest that a full potential of replication, expectedly of the progenitor cell population, is required for the FLASH effect *in vivo*. On the other hand, AsiDNA does not affect the quantity of radiation-induced DNA damage (9). However, by enforcing dividing cells (e.g. stem/progenitor cells) into the G1-phase, it allows the cells to repair before entering into S-phase, increasing the survival of cells that will replace the damaged tissue. Collectively, our results indicate that the activity of AsiDNA™ and FLASH-RT could draw upon dissimilar mechanism interference to result in the same capacity to preserve the progenitor cell population within the targeted organ (i.e. lung, gut). Additional research is in progress to explore the spatio-temporal dynamics of mechanisms leading to radiation-induced pulmonary fibrosis (34).

In summary, we have identified an AsiDNA™-induced reversible G1/S-arrest dependent on the DNA-PK/p53/p21 activation cascade exclusively in healthy normal cells. The activation cascade can be exploited to protect the normal tissue against radiation induced toxicity while maintaining tumour control, thereby acting as a unique bilateral agent.

## Supplementary Material

zcae011_Supplemental_Files

## Data Availability

The scRNA-seq datasets generated from non-irradiated control mice have been deposited in the Gene Expression Omnibus (GEO) repository, with the accession code GSE211713. The scRNA-seq datasets generated from irradiated mice including CONV-RT, AsiDNA CONV-RT and FLASH-RT have been deposited in the Gene Expression Omnibus (GEO) repository, with the code GSE240510.

## Notes

Present addresses: Anouk Sesink, Department of Radiation Oncology, Lausanne University Hospital and Lausanne University, Lausanne, Switzerland.

Present addresses: Margaux Becerra, Valerio Therapeutics, Paris, France.
