# Supplementary material for: The AsiDNA™ decoy mimicking DSBs protects the normal tissue from radiation toxicity through a DNA-PK/p53/p21-dependent G1/S arrest
Source: NAR Cancer. 2024 Mar 12;6(1):zcae011. doi: 10.1093/narcan/zcae011 (PMC10928987; doi:10.1093/narcan/zcae011)
Supplement: zcae011_Supplemental_Files [file zcae011_supplemental_files.zip › Legend Suppl Figure.pdf]

Supplementary Figure 1. Effect of AsiDNA™ on the cell cycle progression of primary and hTERT immortalized normal cells. Cells were incubated with 20 µM and 40 µM of AsiDNA™ for 24 h and 48 h. The percentage of cells in G1, S, and G2/M was analysed by flow cytometry and shown for MRC5 primary fibroblasts (**A**), and VH10-hTERT immortalized fibroblasts (**B**). Data are expressed as mean ± standard deviation (n=3) with significance given by two-way ANOVA, Tukey's multiple comparison test, and represented above the bar plots. BJ primary fibroblasts (**C**) and RPE-hTERT immortalized epithelial cells (**D**) were pulse-labelled with BrdU at the end of AsiDNA treatment. Representative images of the bivariate analysis by flow cytometry of BrdU incorporation versus DNA content (PI) are shown in (**C**) and (**D**). The line across the plots delineates BrdU positive ($BrdU^+$) and negative ($BrdU^-$) cells.

Supplementary Figure 2. No effect of Nol8 treatment on RPE-hTERT cell cycle progression. (**A**) PARylation after AsiDNA™ and Nol8 treatment in various cell lines. (**B**) Gamma-H2AX immunofluorescence post 10 µM AsiDNA™ or Nol8 treatment. Images are taken at 24 h of treatment. (**C**) Representative images of the bivariate analysis by flow cytometry of BrdU incorporation versus DNA content (PI) in RPE-hTERT cells upon Nol8 treatment. (**D**) The percentage of cells in G1, S, and G2/M is shown in RPE-hTERT cells upon Nol8 treatment. Data are expressed as mean ± standard deviation (n=3) with significance given by two-way ANOVA, Tukey's multiple comparison test, and represented above the bar plots. (**E**) Western blot analysis of HSP-90 phosphorylation, p53, p21 and β-actin expression in RPE-hTERT cells upon 20 µM Nol8 treatment for 24 h or 2 h after 5 Gy ionizing radiation (IR).

Supplementary Figure 3. AsiDNA™-induced cell cycle arrest is dependent on DNA-PK, p53, and p21. Mean percentage of BJ cells per cell cycle phase at 48 h of (**A**) AsiDNA™/NU or AsiDNA™/Olaparib, and (**B**) AsiDNA™/siCtr, AsiDNA™/sip53, or AsiDNA™/siDNA-PK. Following the treatments, the cells were stained with PI and analysed by FACS. Data are expressed as mean ± standard deviation (n=3) with significance given by two-way ANOVA test and represented above the bar plots. (**C**) RPE-hTERT cells were exposed to 20 µM AsiDNA, 10 µM p21 inhibitor (UC2288) or a combination of both for 48 h. The percentage of cells in G1, S, and G2/M was analysed by flow cytometry based on PI staining. Mean percentage of RPE-hTERT shp53 cells per cell cycle phase after AsiDNA™ treatment. Data are expressed as mean ± standard deviation (n=3) with significance given by two-way ANOVA,

Tukey's multiple comparison test, and represented above the bar plots. (**D**) Western blot analysis of DNA-PKcs, p53, and p21 expression following transfection of siRNA DNA-PKcs, siRNA p53, and siRNA p21 in RPE-hTERT. #1 and #2 represent independent replicates. (**E**) Western blot analysis of p53 and p21 expression levels in RPE-hTERT cells upon combined 20 µM AsiDNA™ with 10 µM NU7026 for 5 h, 24 h and 48 h. (**F**) Western blot analysis of p53 and p21 expression level in RPE-hTERT cells proficient and deficient for p53 in response to 20 µM AsiDNA™ for 2 4h and 48 h, or 30 min post 10 Gy ionizing radiation (IR). (**G**) Western blot analysis of p53 and p21 expression level in RPE-hTERT $p21^{-/-}$ in response to 20 µM AsiDNA™ for 48 h. In (**D-G**), β-actin was used as a loading control. Gels are representatives of at least 3 independent experiments. NT: no treatment. M.W.: Molecular weight protein markers, upper band: 50 kDa, lower band: 37 kDa. *, p-value < 0.05; **, p-value < 0.01; ***, p-value < 0.001; ****, p-value < 0.0001.

Supplementary Figure 4. Recovery of cell cycle progression after AsiDNA™ treatment. RPE-hTERT cells received increased recovery times post AsiDNA™ treatment. (**A**) Western blot analysis of RPE-hTERT cells of p53 and p21 expression levels. (**B**) Mean percentage of RPE-hTERT cells per cell cycle phase measured by PI staining and analysed by FACS. Data are expressed as mean ± standard deviation (n=3) with significance given by two-way ANOVA, Tukey's multiple comparison test, and represented above the bar plots. *, p-value < 0.05; **, p-value < 0.01; ***, p-value < 0.001; ****, p-value < 0.0001.

Supplementary Figure 5. No p21 induction in AsiDNA™-treated p53 proficient tumour cells and no AsiDNA™-induced G1/S arrest in p53 deficient tumour cells. (**A**) Western blot analysis of p21 induction in A549, HCT116, U-2OS, and RPE-hTERT cells treated with 20 µM AsiDNA for 24 h and 48 h. β-actin was used as loading control. (**B**) Mean percentage of p53-deficient Daoy cells per cell cycle phase at 24 h and 48 h of 20 µM and 40 µM AsiDNA™ treatment. Following the treatments, the cells were stained with PI and analysed by FACS. Data are expressed as mean ± standard deviation (n=3).

Supplementary Figure 6. Representative images of lung fibrosis at 5 months post treatment, stained with H&E and Masson staining for each treatment group of experiment. Arrows indicate visible grade 5 Ashcroft fibrosis score. The images and histopathological analysis were provided by Institut Curie, PMDT-pathologie-PathEx platform. Scale bar = 200 µm.

Supplementary Figure 7. AsiDNA™ treatment did not result in decreased Ki67 signal in intestinal crypts. (**A**) Representative images of intestinal rolls stained with DAPI and Ki67 for each treatment group. (**B**) Ki67 positive cells per 100 detected cells in small intestinal crypts after AsiDNA™ treatment. A total number of 14000-16000 cells per mouse were scored. Scale bar = 50 µm.
