# Supplementary material for: The AsiDNA™ decoy mimicking DSBs protects the normal tissue from radiation toxicity through a DNA-PK/p53/p21-dependent G1/S arrest
Source: NAR Cancer. 2024 Mar 12;6(1):zcae011. doi: 10.1093/narcan/zcae011 (PMC10928987; doi:10.1093/narcan/zcae011)
Supplement: zcae011_Supplemental_Files [file zcae011_supplemental_files.zip › Suppl Tables 1 2 3.pdf]

Supplementary data Table 1: Information in vivo thorax irradiation setup parameters

Mode	CONV	FLASH
Energy (MeV)	5	5
Applicator type (mm diameter)	50	50
Collimator type	Graphite	Graphite
Field dimension (mm^2)	18x26	18x26
Detector type	Gafchromic EBT-XD	Gafchromic EBT-XD
Skin-collimator Distance (mm)	35	35
Depth of measured dose (mm)	0	0
Given dose (Gy)	13.2-16.21	12-14.95
Frequency (Hz)	10	100
Dose rate (Gy/s)	0.12-0.13	399-498
Dose/pulse (Gy/p)	0.0117-0.0130	3.0-3.7
Pulse length measured (µs)	-	2.78-3.25
No. of pulses	1080-1296	4
Treatment time (s)	107.9-129.4	0.03

Supplementary data Table 2: Information in vivo intestine irradiation setup parameters

Mode	CONV	FLASH
Energy (MeV)	6	6
Applicator type (mm diameter)	50	50
Collimator type	Brass	Brass
Field dimension (mm^2)	30x33	30x33
Detector type	Gafchromic EBT-XD	Gafchromic EBT-XD

**Skin-collimator Distance (mm)**	0	0
**Depth of measured dose (mm)**	15	15
**Given dose (Gy)**	10	10
**Frequency (Hz)**	25	300
**Dose rate (Gy/s)**	0.11-0.14	3000
**Dose/pulse (Gy/p)**	4-6 x 10^{-3}	5
**Pulse length measured (μs)**	3.5	3.5
**No. of pulses**	1750-2250	2
**Treatment time (s)**	70-90	0.03

Supplementary data Table 3: Ashcroft fibrosis scoring after thorax irradiation in mice

Mice ID (LL= Left lobe, SL= Superior lobe)	Treatment	Ashcroft fibrosis score	Additional observations	Day of sacrifice	Main reason for the sacrifice
**3293 SL**	NT	0		158	For scRNA-seq
**3294 LL**	NT	0		158	For scRNA-seq
**3295 LL**	NT	0		158	For histology
**3341 SL**	NT	0		200	Final end-point
**3342 LL**	NT	0		200	Final end-point
**3343 LL**	NT	0		200	Final end-point
**4103 LL**	NT	0		200	Final end-point
**4104 LL**	NT	0		200	Final end-point

**4105 LL**	NT	0		200	Final end-point
**3305 LL**	CONV 13 Gy	4-5		142	Ethical end-point
**3306 LL**	CONV 13 Gy	5	ossification	147	Ethical end-point
**3307 LL**	CONV 13 Gy	5		145	Ethical end-point
**3308 SL**	CONV 13 Gy	5		126	Ethical end-point
**3309 LL**	CONV 13 Gy	4-5		158	Ethical end-point
**3310 LL**	CONV 13 Gy	5		138	Ethical end-point
**3317 LL**	CONV 13 Gy AsiDNA	4-5		165	Ethical end-point
**3318 LL**	CONV 13 Gy AsiDNA	5		154	Ethical end-point
**3319 LL**	CONV 13 Gy AsiDNA	4		200	Final end-point
**3320 LL**	CONV 13 Gy AsiDNA	5		132	Ethical end-point
**3321 LL**	CONV 13 Gy AsiDNA	5		142	Ethical end-point
**3322 LL**	CONV 13 Gy AsiDNA	5		149	Ethical end-point
**3306 LL**	CONV 13 Gy AsiDNA	2		200	Final end-point
**3307 LL**	CONV 13 Gy AsiDNA	5		200	Final end-point
**3308 LL**	CONV 13 Gy AsiDNA	4		200	Final end-point
**3353 LL**	FLASH 13 Gy	5		194	Ethical end-point
**3354 LL**	FLASH 13 Gy	5-6		141	Ethical end-point

**3356 LL**	FLASH 13 Gy	5		200	Final end-point
**3357 LL**	FLASH 13 Gy	5		200	Final end-point
**3358 LL**	FLASH 13 Gy	5-6		194	Ethical end-point
**3365 LL**	FLASH 13 Gy AsiDNA	5	many macrophages	183	Ethical end-point
**3366 LL**	FLASH 13 Gy AsiDNA	6		200	Final end-point
**3367 LL**	FLASH 13 Gy AsiDNA	6		194	Ethical end-point
**3368 LL**	FLASH 13 Gy AsiDNA	5-6		158	Ethical end-point
**3369 LL**	FLASH 13 Gy AsiDNA	6		158	Ethical end-point
**3370 LL**	FLASH 13 Gy AsiDNA	6		200	Final end-point

Ashcroft fibrosis score

- Grade 0: normal lung
- Grade 1: small fibrous changes in the alveoli of the lung
- Grade 2: presence of some fibrous changes
- Grade 3: contiguous fibrous modification
- Grade 4: small masses of fibrosis
- Grade 5: confluent fibrotic masses
- Grade 6: large contiguous masses of fibrosis
- Grade 7: confluent masses of fibrosis extending into almost all the parenchyma
- Grade 8: complete obliteration by fibrosis

Number of mice sacrificed per group due to ethical end-point: NT = 0/7; CONV 13 Gy = 6/6; CONV 13 Gy + AsiDNA = 5/9; FLASH 13 Gy = 3/5; FLASH 13 Gy + AsiDNA = 4/6

scRNA-seq: single-cell RNA sequencing
