# Supplementary material for: The AsiDNA™ decoy mimicking DSBs protects the normal tissue from radiation toxicity through a DNA-PK/p53/p21-dependent G1/S arrest
Source: NAR Cancer. 2024 Mar 12;6(1):zcae011. doi: 10.1093/narcan/zcae011 (PMC10928987; doi:10.1093/narcan/zcae011)
Supplement: zcae011_Supplemental_Files [file zcae011_supplemental_files.zip › Supplementary Figures AsiDNA paper 2024_AS.pdf]

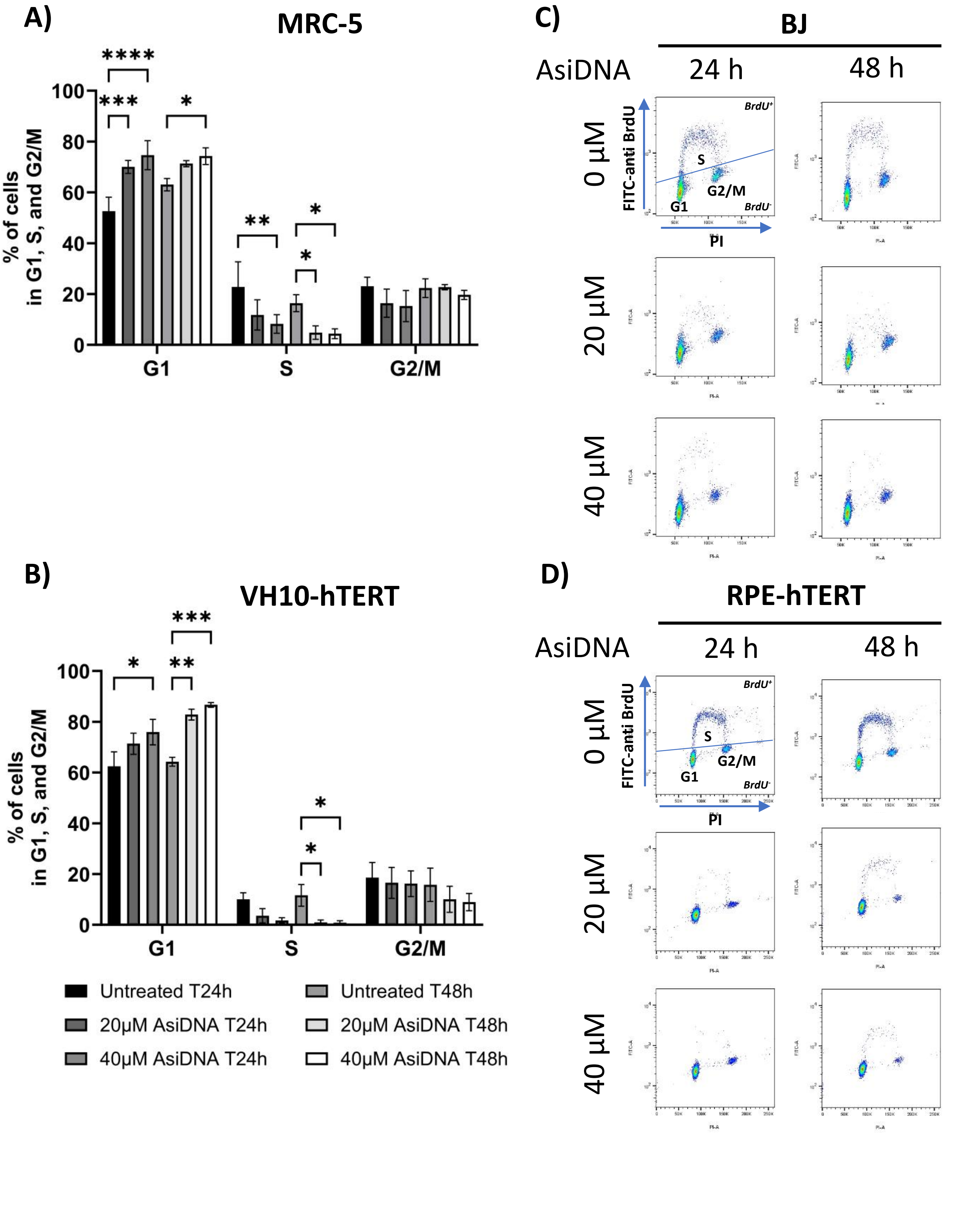

Supplementary Figure 1

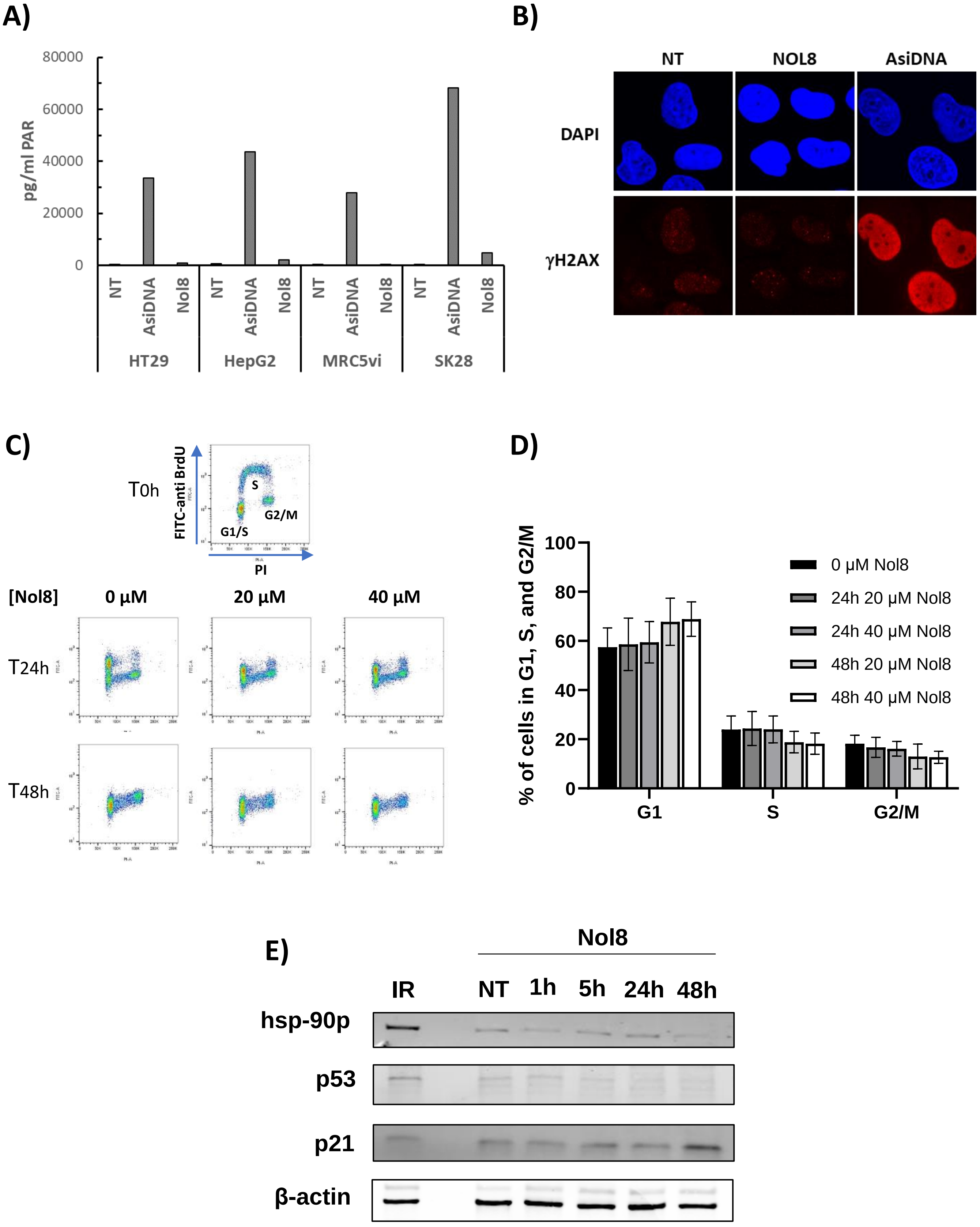

Supplementary Figure 2

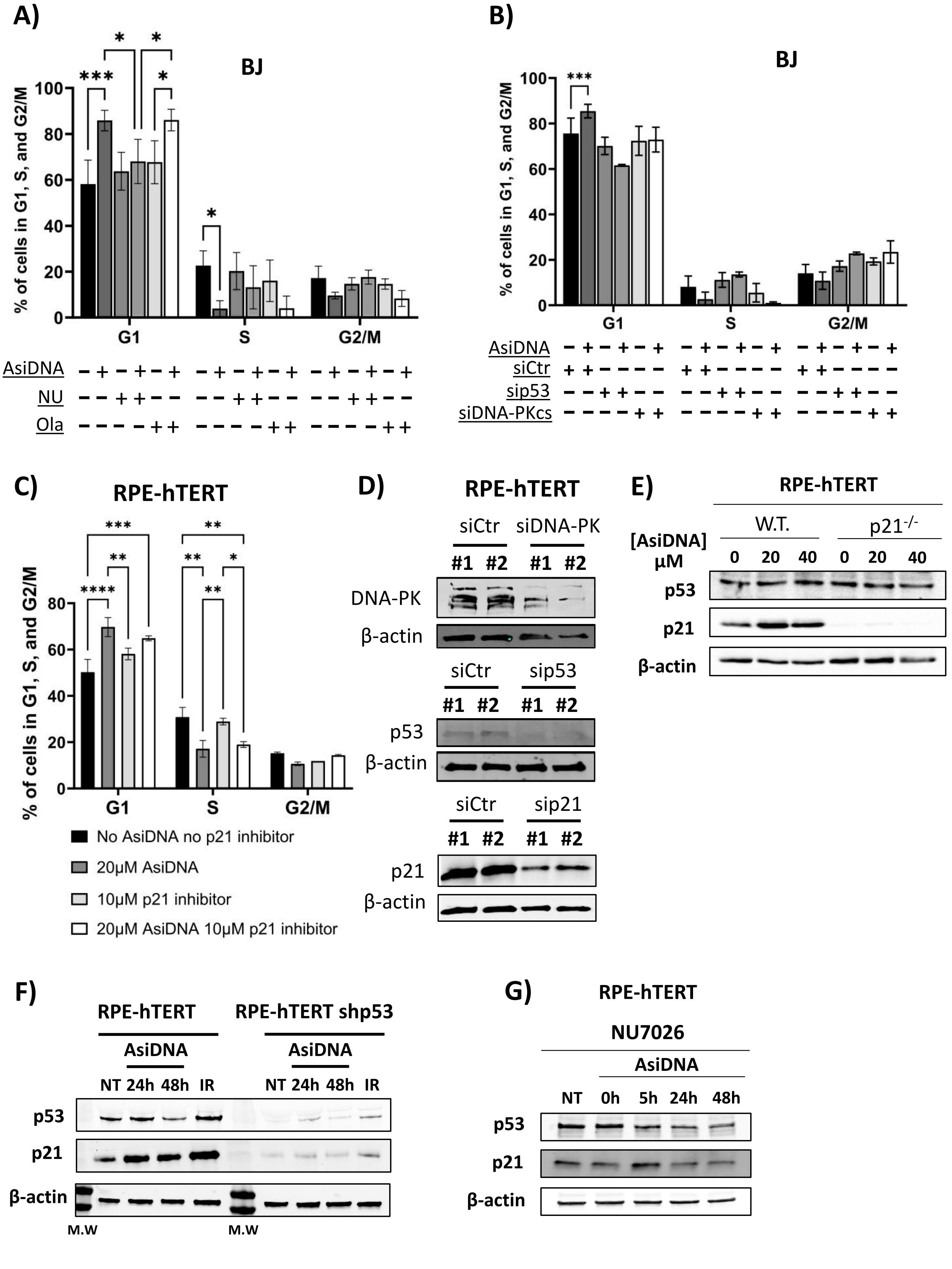

Supplementary Figure 3

A)

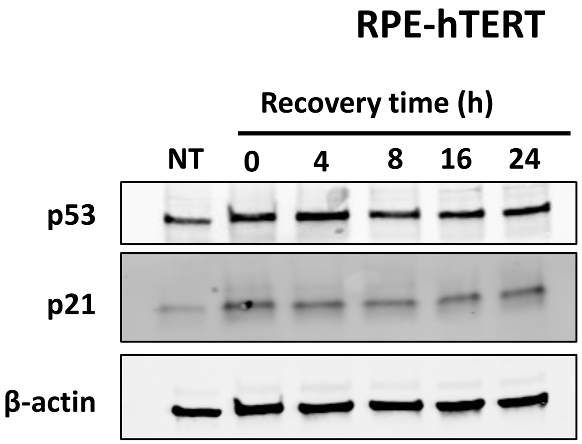

B)

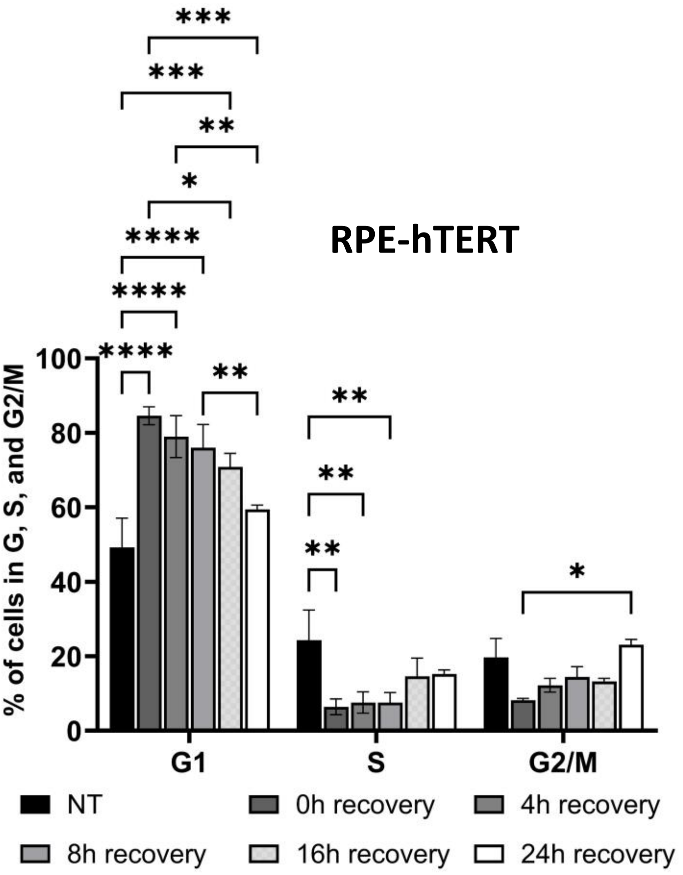

Supplementary Figure 4

A)

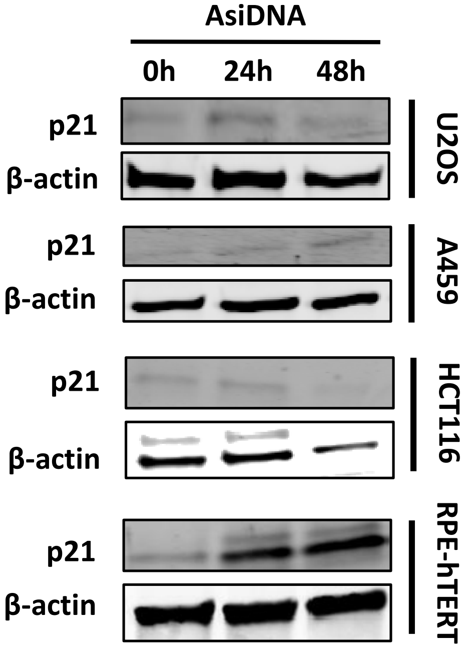

B)

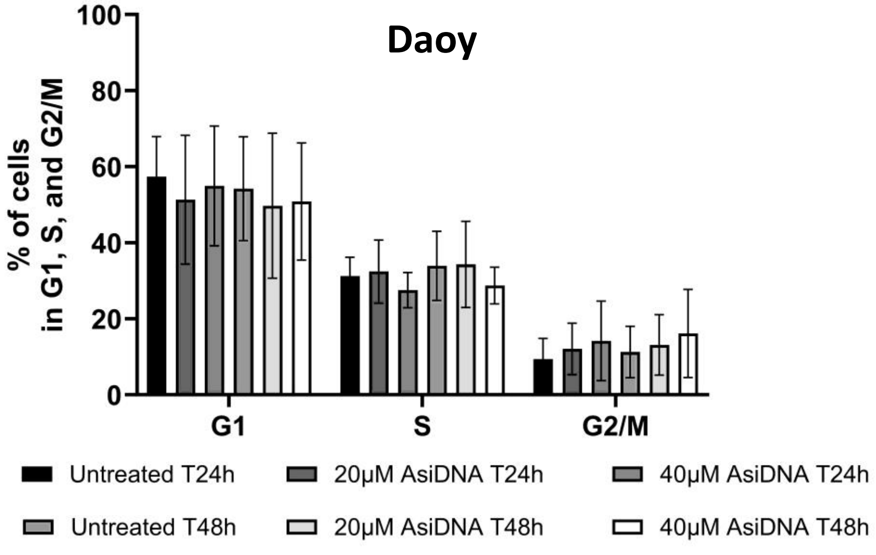

Supplementary Figure 5

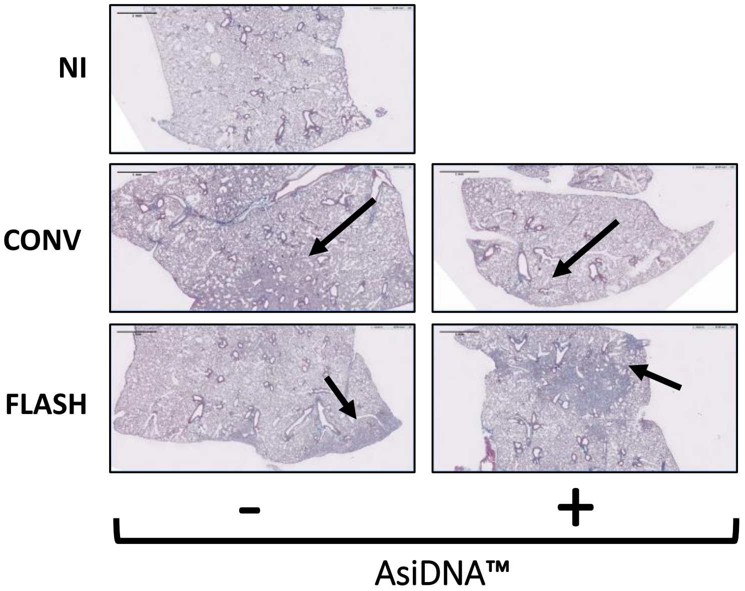

Supplementary Figure 6

A)

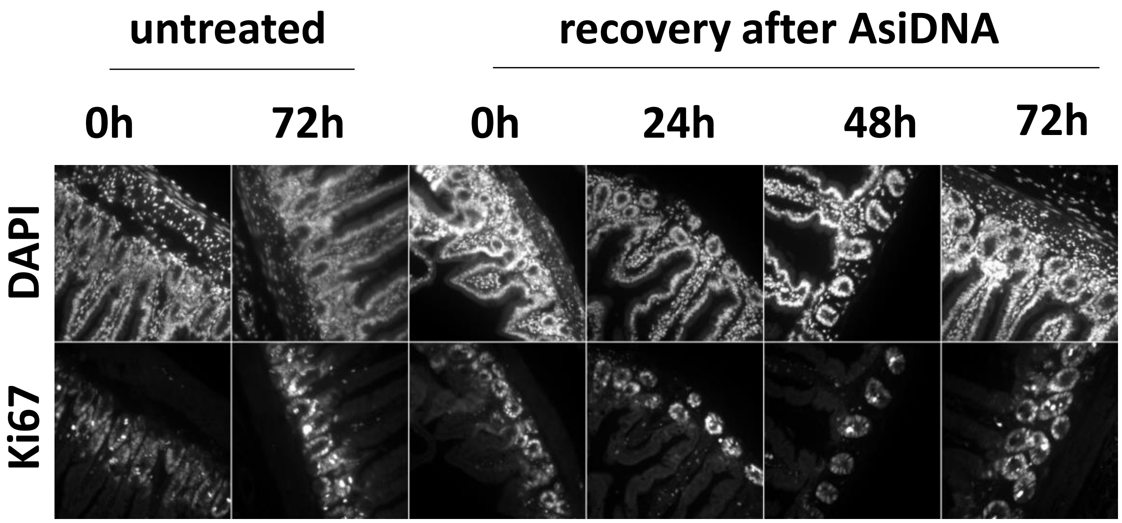

B)

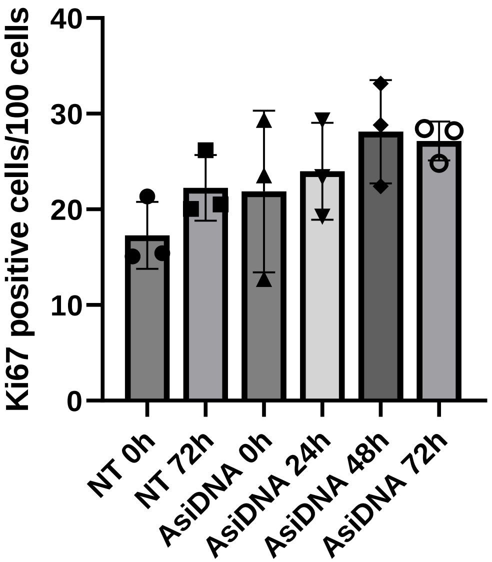

Supplementary Figure 7
