# Supplementary material for: The AsiDNA™ decoy mimicking DSBs protects the normal tissue from radiation toxicity through a DNA-PK/p53/p21-dependent G1/S arrest
Source: NAR Cancer. 2024 Mar 12;6(1):zcae011. doi: 10.1093/narcan/zcae011 (PMC10928987; doi:10.1093/narcan/zcae011)
Supplement: zcae011_Supplemental_Files [file zcae011_supplemental_files.zip › Supplementary M and M.pdf]

PCLS collection

The PCLS were obtained from the lungs of female C57BL/6J mice or male and female C57BL/6J p53 Knock-out mice. Mice were sacrificed by cervical dislocation. Low-melting agarose/medium solution (Invitrogen) of 2,5% was injected into the trachea and the lung lobes were isolated followed by the generation of equal tissue pieces using the surgical punch (8 mm). The isolated tissue is placed in plastic moulds, containing 5% agarose solution. PCLS were generated in DMEM/F-12 medium supplemented with 1% P/S, 1% SVF, 1% L-glutamine and 1% NEAA, using Leica VT 1000 S vibrating blade microtome (Leica Biosystems B.V., Amsterdam, the Netherlands) with a cutting frequency of 90 Hz at position 9 and sectioning speed of 2.25 mm/s at position 9 creating 300 µm-thick lung slices. The slices were placed at 37°C for 30 minutes (to eliminate agarose around the slice) and washed with fresh medium before being transferred to clean culture medium and maintained up to 48h in a humidified incubator in an atmosphere of 5% CO2 at 37°C. AsiDNA™ or Nol8 treatment with 5µM was performed after the additional wash with fresh medium, followed by the supplementation of 10 µM EdU 24h post the start of the AsiDNA™ treatment. The slices are fixed with 4% PFA (Electron Microscopy Sciences) at RT overnight in the dark and permeabilized with Saponin. The EdU detection protocol (647nm) and DAPI staining (0.5 µg/ml) was performed following manufacturer's instructions. EdU positive cells are revealed using EdU DetectPro Imaging kit Imaging (488 or 647nm, BCK-EdUPro-IM647/BCK488-IV-IM-S, Baseclick) and viewed with the Inverted spinning disk-TIRF-FRAP (Nikon) using 300 ms emission and 30 % laser, DAPI (405 nm), 400 ms emission and 70 % laser, x10 objective with 50 stacks of 3 µm. Data analysis was performed using IMARIS with spot function and PRISM software.

MIC-MAQ macro

EdU intestine images were generated using 3D SIM microscopy, 40x magnification. Images were processed in Image J with subtraction of background noise. The masks were generated and established on DAPI-positive cells. Nuclei parameters: cellpose model cyto2, cellpose cell diameter 80, exclude on edges yes, SPOT EdU Parameters, background reduction of 40 pixels, cellpose segmentation on and pixel diameter of 80. Total detected nuclei and EdU-positive nuclei were calculated. Ki67 intestine images were generated and processed as described

above. P21 intestine images were generated using a Zeiss microscope, 20x magnification. Images were processed in Image J by image colour deconvolution, separating nuclei and p21 staining, and image inverting. Additional processing occurred as described above.
